# Early diagnosis of Alzheimer’s disease using machine learning: a multi-diagnostic, generalizable approach

**DOI:** 10.1186/s13195-022-01047-y

**Published:** 2022-08-03

**Authors:** Vasco Sá Diogo, Hugo Alexandre Ferreira, Diana Prata, for the Alzheimer’s Disease Neuroimaging Initiative

**Affiliations:** 1 Instituto de Biofísica e Engenharia Biomédica, Faculdade de Ciências da Universidade de Lisboa, 1749-016 Lisboa, Portugal; 2 Iscte-Instituto Universitário de Lisboa, CIS-Iscte, Lisboa, Portugal; 3 Department of Old Age Psychiatry, Institute of Psychiatry, Psychology and Neuroscience, King’s College London, London, UK

**Keywords:** Alzheimer’s disease, Mild cognitive impairment, Dementia, Early diagnosis, Prognosis, Classification, Machine learning, Graph theory

## Abstract

**Background:**

Early and accurate diagnosis of Alzheimer’s disease (AD) is essential for disease management and therapeutic choices that can delay disease progression. Machine learning (ML) approaches have been extensively used in attempts to develop algorithms for reliable early diagnosis of AD, although clinical usefulness, interpretability, and generalizability of the classifiers across datasets and MRI protocols remain limited.

**Methods:**

We report a multi-diagnostic and generalizable approach for mild cognitive impairment (MCI) and AD diagnosis using structural MRI and ML. Classifiers were trained and tested using subjects from the AD Neuroimaging Initiative (ADNI) database (*n* = 570) and the Open Access Series of Imaging Studies (OASIS) project database (*n* = 531). Several classifiers are compared and combined using voting for a decision*.* Additionally, we report tests of generalizability across datasets and protocols (IR-SPGR and MPRAGE), the impact of using graph theory measures on diagnostic classification performance, the relative importance of different brain regions on classification for better interpretability, and an evaluation of the potential for clinical applicability of the classifier.

**Results:**

Our “healthy controls (HC) vs. AD” classifier trained and tested on the combination of ADNI and OASIS datasets obtained a balanced accuracy (BAC) of 90.6% and a Matthew’s correlation coefficient (MCC) of 0.811. Our “HC vs. MCI vs. AD” classifier trained and tested on the ADNI dataset obtained a 62.1% BAC (33.3% being the by-chance cut-off) and 0.438 MCC. Hippocampal features were the strongest contributors to the classification decisions (approx. 25–45%), followed by temporal (approx. 13%), cingulate, and frontal regions (approx. 8–13% each), which is consistent with our current understanding of AD and its progression. Classifiers generalized well across both datasets and protocols. Finally, using graph theory measures did not improve classification performance.

**Conclusions:**

In sum, we present a diagnostic tool for MCI and AD trained using baseline scans and a follow-up diagnosis regardless of progression, which is multi-diagnostic, generalizable across independent data sources and acquisition protocols, and with transparently reported performance. Rated as potentially clinically applicable, our tool may be clinically useful to inform diagnostic decisions in dementia, if successful in real-world prospective clinical trials.

**Supplementary Information:**

The online version contains supplementary material available at 10.1186/s13195-022-01047-y.

## Introduction

Alzheimer’s disease (AD) is one of the most prevalent health conditions in aging, affecting over 32 million people worldwide [1, 2]. It is a neurodegenerative disease characterized by an initial asymptomatic stage, which occurs about 20 years before symptom onset, and during which neuronal damage takes place [3]. The subsequent early symptomatic stage is characterized by a cognitive decline, which is referred to as “mild cognitive impairment due to AD” (herein referred to as MCI) when the clinicians consider the cognitive decline to be the result of the prodromal stage of AD (as opposed to other types of dementia, medication, depression, or other causes) [4]. As a diagnosis, MCI is useful in predicting future AD, with about 15% of MCI patients converting to AD every year [5]. Although there is no cure for AD, an early diagnosis of the disease is important to start therapies that can slow down its progression and improve its management [6]. However, the diagnosis of MCI is difficult, as cognitive decline is present in healthy aging. This calls for the use of MCI as well as AD diagnostic biomarkers. For both diagnoses, one of the most widely used measures to support the clinical decision is medial temporal atrophy (MTA) as detected by visual inspection of a structural magnetic resonance imaging (MRI) scan [7, 8]. However, this biomarker is not particularly useful for the *early* diagnosis of MCI, as MTA does not occur until late into the disease progression [9]. Other common biomarkers for MCI and AD, such as fluorodeoxyglucose or amyloid positron emission tomography and cerebrospinal fluid measures, are less accessible, more expensive, and more invasive compared to MRI ones [7], which would make the latter more appealing for a first-line, early diagnosis.

Given the limitations of the current diagnostic biomarkers, it is necessary to develop a diagnostic tool that can detect early manifestations and accurately diagnose both MCI and AD. Recently, there have been efforts to develop such a tool using machine learning (ML) methodologies, with structural MRI, which are high-performing in distinguishing “healthy controls (HC) vs. AD” (80–100% accuracy), “MCI vs. AD” (50–85% accuracy), “HC vs. MCI” (60–90% accuracy), and “HC vs. MCI vs. AD” (59–77% accuracy) [10, 11]. Importantly, these studies often use the diagnosis at the time of scanning as the “ground-truth” label to train the ML algorithm, limiting the classifier to be only as good as the clinician. However, clinical accuracy is limited at this initial stage, with 20% of MCI patients transitioning to AD and 16% reverting to normal cognition after approximately 1 year [12]. Therefore, the latest possible diagnosis by the clinician should be used as ground-truth in classifier design, despite in vivo diagnosis still being imperfect even when at later stages (with 10% of patients diagnosed with probable AD not meeting pathological criteria in autopsy [13]). An imprecise ground-truth limits the quality of the statistical model and its clinical usefulness when an early diagnosis is sought. Some studies consider progression from MCI to AD during a follow-up period, differentiating stable from progressive MCI [14]. However, to our knowledge, no study using machine learning for MCI and AD diagnosis has considered other conversions from initial diagnosis, specifically MCI reversion to HC and HC progression to MCI. It is unclear whether studies that consider progression from MCI to AD include subjects with other conversions in their sample and, if so, whether the baseline or the current diagnosis is used as a label. Not including these subjects could inflate the performance of classifiers aiming at future diagnosis and also limits their applicability. Thus, to increase classifier quality, in the current study, we considered all types of transitions and used ground-truth diagnostic labels at a minimum of 1-year follow-up and a maximum of 3-year follow-up.

Importantly, in the present study, we aimed to develop an MRI-based *multi-diagnostic* classification biomarker. This classifier is multi-diagnostic in the sense that it differentiates HC, MCI, and AD simultaneously, which the vast majority of studies that consider HC, MCI, and AD do not [11], and which more closely approximates the decision clinicians face. Furthermore, we used data from multiple datasets and MRI acquisition protocols. Specifically, surpassing a common limitation in the literature, we assessed our classifiers’ generalizability across two publicly available independent datasets [10]. Secondly, we assessed generalizability across the two most common T1-weighted acquisition protocols: a magnetization-prepared rapid gradient echo (MPRAGE) sequence and a spoiled gradient echo with inversion recovery preparation (IR-SPGR) sequence. The acquisition protocol has also been shown to impact brain atrophy rate measurements [15] and image signal-to-noise and contrast-to-noise ratios [16], but its impact on ML classification has not been studied, and, more importantly, different protocols are often used unbalanced between diagnostic groups, which may gravely bias (particularly, inflate) classification performance.

ML-based diagnosis classification biomarkers should stem from a careful trade-off between complexity and interpretability, such that they explore complexity to achieve statistical power but retain pathophysiological interpretability which can be informative and reassuring to the clinician. Most previously published classifiers largely lack interpretability and do not provide any insight into how the classification decision was reached [11, 17]. We exclusively used simple linear ML algorithms and tree-based algorithms, so that feature importances could be extracted from the classifiers, to retain some level of interpretability, while also combining classifiers into a complex ensemble. To further increase complexity, above using raw morphometric structural features extracted from the MRI scan, previous studies have built ML classifiers using structural T1-weighted MRI graph theory (GT) features for AD diagnosis. GT morphometry-derived features allow the classifiers to account for complex inter-dependence between brain regions, thus potentially increasing predictive power. Studies using GT features have obtained a good performance in “HC vs. AD” classification (87.0–92.4% area under the receiver operating characteristic curve (AUC)) [18–21] and in multi-diagnostic “HC vs. MCI vs. AD” classification (72.9% AUC) [18]. However, only one of these studies [21] reported the performance difference between using GT vs. morphometric features, having found that thickness-based GT features result in very modest improvement when compared to raw thickness measures (92.4% from 91.6% AUC, respectively, in “HC vs. AD”). Since GT metrics may reduce classifier interpretability, these should only be used if the improvement obtained outweighs the loss of interpretability. In the present study, we build classifiers with and without GT metrics to determine whether including such metrics is preferable. We used structural MRI GT features which have all been previously associated with AD (degree [22], clustering coefficient [23], node betweenness centrality [24], and eigenvector centrality [25]). However, unfortunately, we could not herein predict the direction of these effects, as they can depend on edge definition [26] and vary across studies [27].

The ML biomarker literature is also rife with unclear reports, which hinders comparability between findings and an insight into their clinical applicability [28]. We aimed to report classifier performance transparently and comprehensively. Along with commonly reported metrics such as sensitivity, specificity, and AUC, we also report more robust metrics such as MCC [29, 30], as well as negative and positive predictive values (NPV and PPV) which are standardized (for better comparability) [31] and prevalence-adjusted (for better clinical context interpretation). We also report performance stratified by sex, which may be relevant in clinical practice, as well as confusion matrices where possible, allowing readers to calculate additional (unreported) metrics. Finally, we sought to evaluate our classifier using our own biomarker evaluation framework published in 2014 [32], to inform on our most inclusive classifiers’ potential clinical applicability.

In sum, we report a ML-based diagnostic tool for MCI and AD which tackles limitations of previous studies, specifically by being (1) multi-diagnostic; (2) trained and tested across 2 independent data sources with multiple acquisition protocols; (3) with tests of generalizability across datasets and protocols, the latter being completely novel in the context of AD; (4) based on baseline scans and a follow-up diagnosis, regardless of progression; (5) with transparently reported performance, and (6) with an evaluation of potential clinical applicability.

## Materials and methods

### Datasets

Data used in the preparation of this article were obtained from the publicly available Alzheimer’s Disease Neuroimaging Initiative (ADNI) database (adni.loni.usc.edu) and the Open Access Series of Imaging Studies (OASIS) project database. The most recent visit in which a diagnosis was made was considered the best available “ground-truth” to train the classifiers. Furthermore, the most recent diagnosis visit must have been at least 1 year after the selected scan for classifier training. The maximum follow-up time was of 3 years. Furthermore, diagnosis transitions must have occurred at least 6 months after the MRI scan. For each subject, we selected their earliest available structural MRI scan that fulfilled our study’s requirements (in the next section). Differences between diagnoses in age and sex (which are known to impact brain structure [33, 34]) and “time from MRI to most recent diagnosis” (which relates positively with the likelihood of diagnostic transitions) were estimated with 1-way ANOVAs (or, if residuals had non-parametric distributions, Mann-Whitney tests) and chi-square.

#### ADNI

The ADNI was launched in 2003 as a public-private partnership, led by principal investigator Michael W. Weiner, MD. The primary goal of ADNI has been to test whether serial MRI, positron emission tomography, other biological markers, and clinical and neuropsychological assessment can be combined to measure the progression of MCI and early AD. From ADNI, 570 subjects were included (211 HC, 188 MCI, 171 AD). To ensure diagnostic criteria equivalence across the different ADNI samples (ADNI, ADNI2, ADNIGO, and ADNI3), besides the diagnosis attributed by the clinician based on a clinical interview and exam results, subjects had to fulfill additional criteria based on the ADNI2 procedures manual. Specifically, HC must have a Mini-Mental State Exam (MMSE) score of at least 24 and a Clinical Dementia Rating (CDR) of 0; MCI patients must have an MMSE score of at least 24 and a CDR of 0.5 with a Memory Box score of at least 0.5; and AD patients must have an MMSE score below 27 and a CDR of at least 0.5.

#### OASIS

From the OASIS-3 dataset of the OASIS project*,* 531 subjects were included (463 HD, 70 AD) [35]. OASIS subjects had to fulfill the same MMSE and CDR requirements used for ADNI subjects and must have been diagnosed by a clinician based on a clinical interview and exam results.

### MRI acquisition

#### ADNI

All ADNI subjects underwent a T1-weighted 3.0 T MRI scan using a MPRAGE (TR = 2300 ms, TE = 2.84–3.25 ms, TI = 900 ms, FA = 8°/9°) or a IR-SPGR (TR = 6.98–7.65 ms, TE = 2.84–3.20 ms, TI = 400 ms, FA = 11°) sequence. Scans using parallel imaging acceleration techniques were excluded because, while some evidence shows that parallel imaging has little impact on brain atrophy rate measurements [15] and morphometric measures [36], differences have been found when using more complex longitudinal measures [37]. As ML methods are more sensitive to peculiarities in the data, acceleration could impact classifier performance. Out of 864 subjects in the ADNI dataset with 3.0 T MRI scan and a follow-up time of at least 1 year, 294 were excluded due to inclusion criteria for acquisition parameters, leaving the final sample of 570 subjects.

#### OASIS

All OASIS subjects underwent a T1-weighted 3.0 T MRI scan (TR = 2400 ms, TE = 3.16 ms, TI = 1 s, FA = 8°) using an MPRAGE sequence with a parallel imaging acceleration in-plane factor of 2 (not excluded as all acquisitions used parallel imaging).

### MRI analysis

#### Morphometric features

MRI preprocessing was performed using the FreeSurfer v6.0.0 standard pipeline [38]. The cortical structure of each hemisphere was parcellated into 34 regions of interest (ROIs). For each ROI, 8 measures were obtained (surface area, volume, average cortical thickness, standard deviation of the cortical thickness, integrated rectified mean curvature, integrated rectified Gaussian curvature, folding index, and intrinsic curvature index). Additionally, volumes were extracted from 40 subcortical ROIs and 64 white matter ROIs. This set of measures was decided a priori because they reflect morphometric alterations associated with MCI and AD, specifically brain atrophy [39], cortical thinning [40], cortical gyrification patterns [41], and white matter changes [42]. Finally, each hippocampus was segmented into 13 subfields [43], with 26 additional volumes being obtained. These hippocampal subfield volumes were extracted because this region has been extensively associated with AD and its progression [44]. Overall, 694 structural features were extracted.

#### Graph theory features

GT features were derived from the morphometric data. ROI volumes (209 features) were used to compute a binary graph. The edges of the graph were calculated with the following ratio:

$${Ratio}_{ij}=\frac{2}{\frac{Feature_i}{Feature_j}+\frac{Feature_j}{Feature_i}}$$

Four different thresholds were used for graph binarization (0.3, 0.5, 0.7, and 0.9). From each subject’s graph, 4 node-wise measures were derived: degree (the number of nodes connected to a given node), clustering coefficient (the fraction of a node’s neighbors that are neighbors of each other), node betweenness centrality (the fraction of all shortest paths in the network that contain a given node), and eigenvector centrality (a measure of the influence of a node in a network). These measures were calculated using the Brain Connectivity Toolbox — comprehensive details about these measures can be found in the article accompanying the toolbox [45]. Overall, 836 GT-based features were computed for each subject at each threshold.

### Machine learning classification

**Fig. 1:**
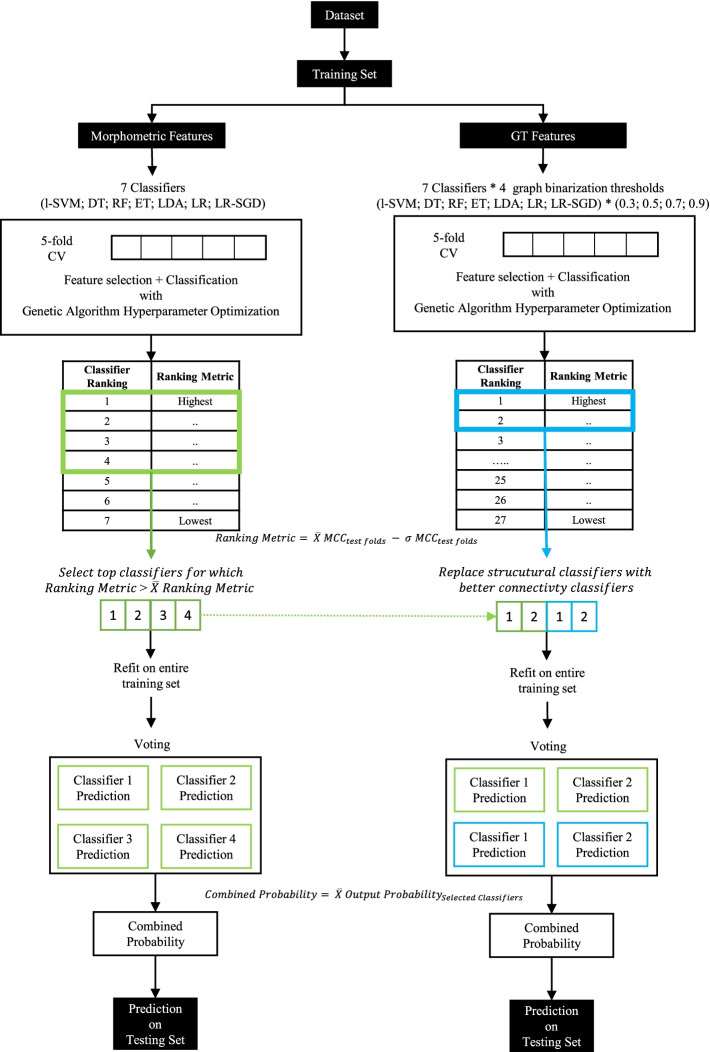
Classification approach for binary classifiers. See the “Machine learning classification” section for a detailed explanation. l-SVM, linear support vector machine; DT, decision tree; RF, random forest; ET, extremely randomized tree; LDA, linear discriminant analysis; LR, logistic regression; LR-SGD, logistic regression with stochastic gradient descent learning; MCC, Matthews correlation coefficient; CV, cross-validation; X̄, average; σ, standard deviation. Green color indicates classifiers using only structural features and blue indicates classifiers using only GT features

**Fig. 2:**
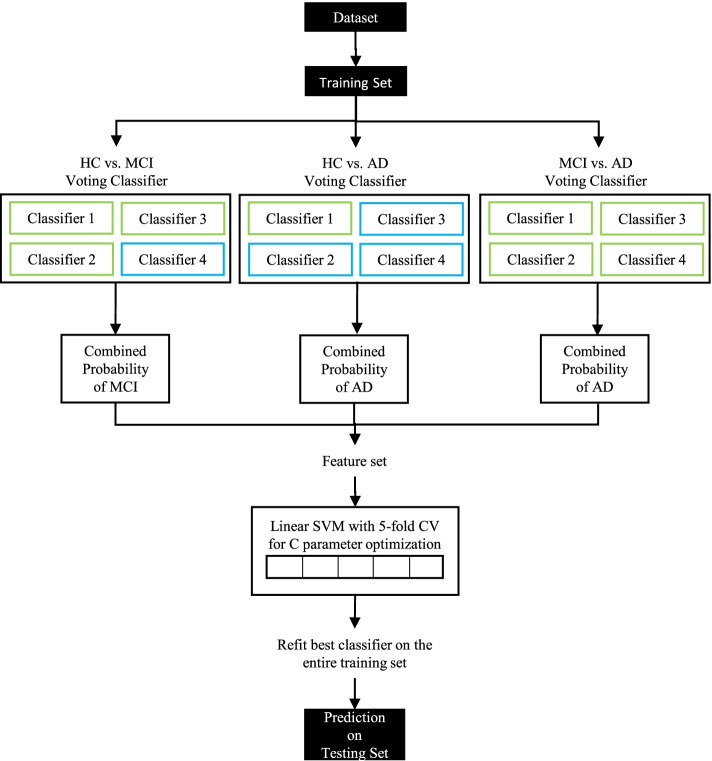
Classification approach for multi-class classifiers. See the “Machine learning classification” section for a detailed explanation. CV, cross-validation; SVM, support vector machine. Green color indicates classifiers using only structural features and blue indicates classifiers using only GT features

The proposed method for multi-diagnostic classification of HC, MCI, and AD is an ensemble of 3 binary classifiers. The approach for each binary classifier and their combination is illustrated in Figs. 1 and 2, respectively.

#### Binary classifiers for morphometric data

For each binary classifier, data was split into training (70%) and test (30%) sets in a stratified fashion, and 7 different classifiers were trained: (1) a linear support vector machine (l-SVM), (2) a decision tree (DT), (3) a random forest (RF), (4) an extremely randomized tree (ET), (5) a linear discriminant analysis classifier (LDA), (6) a logistic regression classifier (LR), and (7) a logistic regression classifier with stochastic gradient descent learning (LR-SGD). These algorithms were chosen for exposing feature importances and outputting probabilities. Feature importance was determined from the weight assigned to the features, except for tree methods (DT, RF, ET), for which it was determined based on impurity. Overall feature importances for the binary classifier were calculated by averaging the feature importances of each classifier voting in that binary decision.

During training, for feature selection and classifier parameter tuning, data was split into 5 stratified cross-validation (CV) folds, meaning 20% of the dataset was iteratively used for the testing and the remaining 80% for training. Within each fold, features were scaled between 0 and 1, and a percentile of best features (between 0 and 100% in steps of 10%) was selected using mutual information, ANOVA *F*-values, or chi-square statistics, and then the classifier was fit. A simple evolutionary algorithm [46] was used to select the best feature percentile, feature selection metric, and classifier hyper-parameters (tested hyper-parameters for each algorithm are in Supplementary Information 1). The evolutionary algorithm evolved over 10 generations, with 10 individuals per generation, a gene mutation probability of 30%, and a crossover probability of 50%, with a tournament size of 3. These parameters were selected based on preliminary tests run on a subset of the ADNI MPRAGE training set. We changed two of these hyper-parameters from their default values. First, we lowered the population size from 50 to 10, because our overall population size was relatively low, and also because this improved training speed. We also increased the gene mutation probability from 10 to 30% as we noticed classifiers were not converging after 10 generations or were getting stuck at local minima. The evolutionary algorithm was optimized for the MCC metric, which is more robust than more commonly used metrics such as accuracy or AUC [29, 30]. The 7 binary classifiers were ranked based on their performance in the training set folds, according to a custom ranking metric which selects for classifiers that have good performance and low variability across folds (mean MCC across folds minus the standard deviation of MCC across folds). Classifiers performing above the mean of the 7 classifiers were refitted on the entire training dataset using the tuned hyper-parameters. Finally, the selected classifiers were combined into a single one by simple voting.

#### Binary classifiers for GT data

The training of classifiers using GT data followed the same approach explained in the previous section, except that those 7 classifiers were trained for each graph-binarization threshold, for a total of 28 classifiers. Since GT features are harder to interpret, we only included GT-based classifiers in the voting if improvement was expected based on their performance (i.e., if they ranked above the selected morphometric-only based classifiers, replacing whichever of the selected morphometric-only classifiers were worst.)

#### Multi-diagnostic classifiers

To combine the output from the binary classifiers, we fitted a linear SVM classifier using a one-vs-one multi-diagnostic strategy on the higher-class probabilities output by each binary. We optimized the C parameter using a 5-fold stratified CV approach and refitted the classifier on the entire training set before testing. Overall feature importance for the multi-diagnostic classifier was calculated by weighting the feature importances of each binary classifier by the coefficient of the linear SVM which combines the binary classifiers.

#### Classifier performance evaluation

All reported performances are those of the test set. We opted to use a test set instead of a CV scheme because cross-validation cannot be used to estimate performance in all of our experiments, as for some experiments the training set data has different characteristics from the testing set data (e.g., in experiment A3, the training set is ADNI MPRAGE and the testing set is ADNI IR-SPGR, meaning there is only one possible split into training and testing, i.e., all ADNI MPRAGE being in the training set and all ADNI IR-SPGR being in the testing set). The test sets were age-matched across diagnosis groups, to ensure that any learning bias introduced by age would not be reflected in our test metrics. Furthermore, the test sets were split evenly between sexes to ensure that sex proportions in the training set would not inflate test results and to allow us to report data stratified by sex with the same confidence interval for both sexes. Finally, the test set had the same proportions of transitions as the training set (i.e., 30% of cases from each type of transition were assigned to the test set). 95% confidence intervals for each metric were estimated using 2000 bootstrapped samples of the test set. Similarly, to compare classifiers’ performance, two-tailed *p*-values were estimated using 2000 bootstrapped samples from the test set of each of the classifiers being compared, with statistical significance threshold set at 0.05. Balanced accuracy (BAC) was calculated as the average of recall obtained on each class. As PPV and NPV should be adjusted for the prevalence of the disease in the diagnostic tests to be useful in the clinical context [47], we report both using prevalence estimates from the first visit in the clinical setting (HC = 42.0%, MCI = 18.6%, AD = 26.3%, non-AD cognitive impairment = 13.1%) [48] and, additionally, at 50% prevalence as “*standardized* predictive values” for easier technical comparison with other predictive tests, as recommended [31].

### Experiments

#### Using ADNI: generalizability across acquisition protocols

Using the ADNI dataset, we focused specifically on the impact of MPRAGE and IR-SPGR protocols on classifier performance. Five diagnostic classification tasks were performed on different subsets of the ADNI dataset (i.e., experiments A1–A5): (A1) MPRAGE only, (A2) IR-SPGR only, (A3) training on MPRAGE and testing on IR-SPGR, (A4) training on IR-SPGR and testing on MPRAGE, and (A5) full ADNI dataset. Of the 570 ADNI subjects, 423 had MPRAGE scans (149 HC, 145 MCI, 129 AD) and 152 had IR-SPGR scans (62 HC, 48 MCI, 42 AD). For classifiers using both types of scans, only the MPRAGE scan is used for the 5 subjects with both scan types. For classifiers using both datasets in training and testing, MPRAGE and IR-SPGR proportions were balanced across diagnosis to prevent the classifier from extrapolating diagnosis information from diagnosis irrelevant characteristics associated with the acquisition protocol. Experiments A6–A10 are the corresponding classifiers that received both morphometric and morphometric-derived GT metrics as inputs. Finally, a classifier was trained for the distinction between MPRAGE and IR-SPGR scans.

#### Using ADNI and OASIS: generalizability across datasets

The second set of experiments focused on the combination of the ADNI and OASIS datasets. Since the OASIS scanning protocol parameters were most similar to ADNI MPRAGE, we only used ADNI MPRAGE scans on this set of experiments. Five diagnostic classification tasks were performed on different combinations of the 2 datasets using morphometric features (i.e., experiments B1–B5): (B1) ADNI MPRAGE only, (B2) OASIS only, (B3) training on ADNI MPRAGE and testing on OASIS, (B4) training on OASIS and testing on ADNI MPRAGE, and (B5) training and testing on both OASIS and ADNI MPRAGE. As above, for classifiers using both datasets in training, the dataset was balanced across diagnosis. Experiments B6–B10 correspond to the classifiers that received both morphometric and morphometric-derived GT metrics as inputs. Additionally, a classifier was trained for the distinction between the OASIS and ADNI scans. A summary of all diagnostic classification experiments is shown in Table 1.

**Table 1:** Summary of diagnostic classification experiments

Experiment	Training set	Testing set	GT metrics
**Using ADNI: testing generalizability across acquisition protocols**			
**A1**	ADNI MPRAGE	ADNI MPRAGE	No
**A2**	ADNI IR-SPGR	ADNI IR-SPGR	No
**A3**	ADNI MPRAGE	ADNI IR-SPGR	No
**A4**	ADNI IR-SPGR	ADNI MPRAGE	No
**A5**	ADNI IR-SPGR and ADNI MPRAGE	ADNI IR-SPGR and ADNI MPRAGE	No
**A6**	ADNI MPRAGE	ADNI MPRAGE	Yes
**A7**	ADNI IR-SPGR	ADNI IR-SPGR	Yes
**A8**	ADNI MPRAGE	ADNI IR-SPGR	Yes
**A9**	ADNI IR-SPGR	ADNI MPRAGE	Yes
**A10**	ADNI IR-SPGR and ADNI MPRAGE	ADNI IR-SPGR and ADNI MPRAGE	Yes
**Using ADNI and OASIS: testing generalizability across datasets**			
**B1**	ADNI MPRAGE	ADNI MPRAGE	No
**B2**	OASIS (MPRAGE)	OASIS (MPRAGE)	No
**B3**	OASIS (MPRAGE)	ADNI MPRAGE	No
**B4**	ADNI MPRAGE	OASIS (MPRAGE)	No
**B5**	ADNI MPRAGE and OASIS (MPRAGE)	ADNI MPRAGE and OASIS (MPRAGE)	No
**B6**	ADNI MPRAGE	ADNI MPRAGE	Yes
**B7**	OASIS (MPRAGE)	OASIS (MPRAGE)	Yes
**B8**	OASIS (MPRAGE)	ADNI MPRAGE	Yes
**B9**	ADNI MPRAGE	OASIS (MPRAGE)	Yes
**B10**	ADNI MPRAGE and OASIS (MPRAGE)	ADNI MPRAGE and OASIS (MPRAGE)	Yes

‘GT metrics’ refers to whether graph theory metrics were given as an input to classifiers, regardless of whether the classifier selected them or not. For each of the experiments A1 through A10, 4 classifiers were built (“HC vs. MCI”; “HC vs. AD”; “MCI vs. AD”; and “HC vs. MCI vs. AD”). For each of the experiments B1 through B10, only 1 classifier was built (“HC vs. AD”, as OASIS did not have MCI subjects available)

#### Diagnosis transition

Since we have follow-up data for all patients, we describe the ground-truth either as “stable” (meaning the diagnosis has not changed between the time of scanning and the most recent clinical follow-up) or as a “transition” (meaning the clinical diagnosis at the time of scanning is different from the most recent clinical follow-up available) and compare performance between “stable” patients and those who “transitioned” within the follow-up time. We did not exclude any transition cases, including diagnosis regression, to avoid artificially inflating model performance, which could arise from only including reliable or progressive diagnosis.

#### Clinical applicability potential

We sought to evaluate the potential for clinical applicability of our most inclusive diagnosis-wise and protocol-wise biomarker without GT features (“HC vs. MCI vs. AD”; experiment A5) and our most inclusive dataset-wise biomarker without GT features (“HC vs. AD”; experiment B5), as a first step towards informing on their potential clinical usefulness. For this, we utilized our own biomarker evaluation framework [32], which takes into account two dimensions, “Quality of Evidence,” which refers to the biomarker’s statistical strength and reproducibility, and “Effect Size,” which refers to its predictive power.

## Results

### Cohort characteristics

The demographic and diagnostic data of the study’s subjects are shown in Table 2. In the ADNI dataset, there was a statistically significant difference between diagnoses in age at the time of scanning (*F*(2, 570) = 4.19, *p* = 0.016) such that AD subjects were older than HC (*t*(382) = 2.88, *p* = 0.004), with no significant difference between MCI and AD (*t*(359) = 1.87, *p* = 0.063) or MCI and HC subjects (*t*(399) = 0.888, *p* = 0.375), and in sex between diagnosis (*X*^2^(2, *N* = 570) = 8.18, *p* = 0.017). There was no statistically significant difference in “time from MRI to most recent diagnosis” between diagnoses (*F*(2, 570) = 2.84, *p* = 0.059). In the OASIS dataset, there was a significant difference between diagnoses in age (*U* = 6545, *p* < .001), such that AD subjects were older than HC, and in “time from MRI to most recent diagnosis” (*U* = 9827, *p* < .001) such that HC had a longer follow-up time than AD, but none in sex (*X*^2^(1, *N* = 506) = 2.37, *p* = 0.124).

**Table 2:** Descriptive statistics of demographic and clinical variables

Dataset	Diagnosis	***N***	Sex (M/F)	Age (at MRI scanning)		Time from MRI to the most recent diagnosis (months)	
				Mean	Standard deviation	Mean	Standard deviation
ADNI	**HC**	211	98/113	72.4	6.6	33.6	5.5
	HC stable	186	86/100	73.1	6.3	33.3	5.7
	MCI transition to HC	26	13/12	67.8	7.3	36.0	0.0
	**MCI**	188	113/75	73.1	7.5	31.3	7.9
	MCI stable	172	102/70	72.5	7.4	31.4	7.9
	HC transition to MCI	16	11/5	78.9	5.7	31.1	8.0
	**AD**	171	97/74	74.6	7.9	26.2	9.6
	AD stable	90	50/40	75.2	7.9	20.2	7.9
	MCI transition to AD	83	34/47	73.9	7.9	32.8	6.5
OASIS	**HC**	439	179/260	67.4	8.1	34.6	4.2
	**AD**	67	34/33	75.6	7.5	30.2	7.9

*M* male, *F* female

### ADNI: MPRAGE vs. IR-SPGR

#### Classifier performance

Results from all ADNI MPRAGE vs. IR-SPGR diagnostic experiments with only morphometric features being inputted are presented in Table 3. (Results from the corresponding classifiers being inputted morphometric and GT features can be found in Supplementary Table 1.) All classifiers that received GT features as input alongside morphometric features (experiments A6–A10) made use of GT features for classification. Despite being selected, the use of GT features had no statistically significant impact on performance in any experiment (*p* = 0.060–0.974). The classifier distinguishing between MPRAGE and IR-SPGR scans only selected morphometric features and yielded nearly perfect testing performance (0.968 MCC [CI 95%: 0.914–1.000]; 99.2% BAC [CI 95%: 97.9–100.0%]). Regarding sex, the performance of our most global classifier (experiment A5, HC vs. MCI vs. AD) for females (*N* = 82) was 0.454 MCC [CI 95%: 0.300–0.611] and for males (*N* = 82) was 0.424 MCC [CI 95%: 0.268–0.568], and the difference between sexes was not significantly different (*p* = 0.778).

**Table 3:** Classifier performance using only morphometric features for classifiers using ADNI subjects with IR-SPGR or MPRAGE scans

Experiment	Training set	Testing set	Classification task	MCC [CI: 95%]	BAC [CI: 95%]	ROC AUC [CI: 95%]	Sens [CI: 95%]	Spec [CI: 95%]	PPV (prevalence) [CI: 95%]	NPV (prevalence) [CI: 95%]	PPV (standard) [CI: 95%]	NPV (standard) [CI: 95%]	TN	FP	FN	TP	MCC *p*-value (vs. w/ GT)
A1	ADNI MPRAGE (*N*=295)	ADNI MPRAGE (*N*=128)	HC vs. MCI	0.056 [−0.159;0.259]	52.8% [42.1%; 62.9%]	58.2% [46.5%; 70.1%]	50.0% [35.0%; 64.6%]	55.6% [41.5%; 69.6%]	33.3% [21.0%; 48.5%]	71.5% [59.0%; 81.6%]	53.0% [37.4%; 68.0%]	52.7% [39.0%; 66.3%]	25	20	22	22	0.060
			HC vs. AD	0.814 [0.679; 0.927]	90.8% [84.1%; 96.2%]	97.3% [93.6%; 99.6%]	94.9% [87.2%; 100.0%]	86.7% [76.2%; 95.5%]	81.7% [69.6%; 93.3%]	96.4% [90.5%; 100.0%]	87.7% [78.6%; 95.7%]	94.4% [85.6%; 100.0%]	39	6	2	37	0.974
			MCI vs. AD	0.588 [0.424; 0.751]	79.0% [70.7%; 87.5%]	87.9% [79.9%; 95.3%]	89.7% [78.6%; 97.7%]	68.2% [54.3%; 82.4%]	80.0% [70.9%; 88.7%]	82.4% [64.2%; 96.2%]	73.8% [63.2%; 84.7%]	86.9% [71.7%; 97.3%]	30	14	4	35	0.627
			HC vs. MCI vs. AD	0.350 [0.227; 0.480]	57.7% [50.2%; 65.2%]	76.6% [70.8%; 82.3%]	n/a	n/a	n/a	n/a	n/a	n/a	n/a	n/a	n/a	n/a	0.674
A2	ADNI IR-SPGR (*N*=106)	ADNI IR-SPGR (*N*=46)	HC vs. MCI	0.087 [−0.267; 0.457]	53.8% [38.7%; 70.4%]	69.2% [51.2%; 86.0%]	28.6% [7.1%; 54.5%]	78.9% [61.1%; 95.0%]	37.5% [7.5%; 82.8%]	71.4% [59.8%; 82.5%]	57.5% [15.4%; 91.6%]	52.5% [39.7%; 67.6%]	15	4	10	4	0.860
			HC vs. AD	0.607 [0.313; 0.867]	79.4% [65.0%; 92.9%]	93.5% [82.7%; 100.0%]	69.2% [44.4%; 92.9%]	89.5% [75.0%; 100.0%]	80.5% [52.6%; 100.0%]	82.3% [68.3%; 95.7%]	86.8% [64.0%; 100.0%]	74.4% [57.4%; 93.4%]	17	2	4	9	0.384
			MCI vs. AD	0.408 [−0.028; 0.742]	70.1% [51.5%; 86.7%]	77.5% [55.1%; 94.0%]	61.5% [33.3%; 86.7%]	78.6% [50.0%; 100.0%]	80.3% [48.5%; 100.0%]	59.1% [34.6%; 84.2%]	74.2% [40.0%; 100.0%]	67.1% [42.8%; 88.3%]	11	3	5	8	0.737
			HC vs. MCI vs. AD	0.263 [0.054; 0.491]	49.8% [37.1%; 62.9%]	77.1% [66.4%; 87.5%]	n/a	n/a	n/a	n/a	n/a	n/a	n/a	n/a	n/a	n/a	0.996
A3	ADNI MPRAGE (*N*=423)	ADNI IR-SPGR (*N*=147)	HC vs. MCI	0.268 [0.093; 0.423]	61.9% [54.2%; 69.4%]	73.5% [62.9%; 82.7%]	88.4% [77.8%; 97.4%]	35.5% [23.9%; 48.3%]	37.8% [31.2%; 45.5%]	87.4% [70.8%; 97.7%]	57.8% [50.6%; 65.3%]	75.4% [51.8%; 94.9%]	22	40	5	38	0.816
			HC vs. AD	0.745 [0.612; 0.867]	87.6% [80.7%; 94.0%]	96.3% [92.7%; 98.9%]	88.1% [77.8%; 97.2%]	87.1% [77.9%; 95.1%]	81.0% [68.8%; 92.5%]	92.1% [84.9%; 98.2%]	87.2% [77.9%; 95.2%]	88.0% [77.8%; 97.1%]	54	8	5	37	0.367
			MCI vs. AD	0.647 [0.480; 0.805]	82.4% [74.1%; 90.1%]	87.4% [79.8%; 90.1%]	83.3% [71.4%; 93.8%]	81.4% [68.9%; 92.9%]	86.4% [76.5%; 94.9%]	77.5% [63.0%; 91.3%]	81.7% [69.7%; 92.9%]	83.0% [70.7%; 93.7%]	35	8	7	35	0.496
			HC vs. MCI vs. AD	0.424 [0.312; 0.533]	61.7% [54.6%; 68.8%]	81.6% [75.6%; 87.3%]	n/a	n/a	n/a	n/a	n/a	n/a	n/a	n/a	n/a	n/a	0.618
A4	ADNI IR-SPGR (*N*=147)	ADNI MPRAGE (*N*=423)	HC vs. MCI	0.178 [0.074; 0.287]	57.1% [53.0%; 61.7%]	65.6% [59.5%; 72.0%]	26.9% [20.4%; 34.1%]	87.2% [81.9%;92.3%]	48.2% [33.3%; 66.2%]	72.9% [69.9%; 76.0%]	67.8% [53.0%; 81.6%]	54.4% [50.7%; 58.3%]	##	19	##	39	0.462
			HC vs. AD	0.711 [0.631; 0.792]	85.4% [81.2%; 89.4%]	93.5% [90.7%; 95.9%]	82.2% [75.0%; 88.1%]	88.6% [83.3%; 93.3%]	81.9% [73.8%;89.2%]	88.8% [84.2%; 92.6%]	87.8% [81.8%; 92.9%]	83.3% [76.9%; 88.7%]	##	17	23	##	0.893
			MCI vs. AD	0.454 [0.353; 0.551]	71.7% [66.6%; 76.5%]	81.4% [76.2%; 86.3%]	87.6% [81.4%; 92.9%]	68.3% [56.5%; 70.9%]	73.7% [68.9%; 78.5%]	76.16% [64.6%; 86.4%]	66.5% [61.0%; 72.0%]	81.8% [72.1%; 90.0%]	81	64	16	113	0.114
			HC vs. MCI vs. AD	0.372 [0.307; 0.431]	57.0% [53.4%; 60.3%]	75.5% [72.2%; 78.8%]	n/a	n/a	n/a	n/a	n/a	n/a	n/a	n/a	n/a	n/a	0.492
A5	ADNI IR-SPGR and ADNI MPRAGE (*N*=379)	ADNI IR-SPGR and ADNI MPRAGE (*N*=164)	HC vs. MCI	0.265 [0.085; 0.441]	62.6% [54.0%; 71.0%]	64.4% [53.7%; 74.1%]	47.2% [34.5%; 60.4%]	78.0% [66.7%; 87.7%]	48.7% [31.5%; 68.5%]	76.9% [69.7%; 83.3%]	68.2% [50.9%; 83.1%]	59.6% [50.5%; 68.9%]	46	13	28	25	0.404
			HC vs. AD	0.789 [0.669; 0.892]	89.5% [83.7%; 94.7%]	96.0% [92.3%; 98.8%]	94.2% [87.0%; 100.0%]	84.7% [75.4%; 93.8%]	79.4% [68.9%; 91.0%]	95.9% [90.3%; 100.0%]	86.0% [78.0%; 94.2%]	93.6% [85.3%; 100.08%]	50	9	3	49	0.883
			MCI vs. AD	0.592 [0.433; 0.734]	79.1% [71.0%; 86.2%]	88.1% [80.6%; 93.6%]	88.5% [79.2%; 96.2%]	69.8% [55.9%; 81.1%]	80.6% [71.8%; 87.8%]	81.1% [65.5%; 93.8%]	74.6% [64.2%; 83.6%]	85.9% [72.9%; 95.5%]	37	16	6	46	0.774
			**HC vs. MCI vs. AD**	**0.438** **[0.330;0.541]**	**62.1%** **[55.5%; 68.2%]**	**77.7%** **[72.2%; 82.6%]**	**n/a**	**n/a**	**n/a**	**n/a**	**n/a**	**n/a**	**n/a**	**n/a**	**n/a**	**n/a**	**0.505**

MCC *p*-value refers to the *p*-value for the MCC metric for the comparison with the equivalent classifier (i.e., same training and test sets) with morphometric and GT features used as input. PPV/NPV “prevalence” are calculated with an MCI prevalence of 30.7% for the “HC vs. MCI” classifiers; an AD prevalence of 38.5% for the “HC vs. AD” classifiers; and an AD prevalence of 58.6% for the MCI vs. AD classifier (these correspond to the relative prevalence of the positive class based on prevalence estimates from the first visit in the clinical setting of 42.0% for HC, 18.6% for MCI, and 26.3% for AD) [48]. PPV/NPV “standard” are calculated with a prevalence of 50% to allow comparison with other studies

*Legend*: *CI* confidence interval, *MCC* Matthew’s correlation coefficient, *ROC AUC* area under the receiver operating characteristic curve, *BAC* balanced accuracy, *Sens* sensitivity, *Spec* specificity, *PPV* positive predict value, *NPV* negative predictive value, *TN* true negatives, *FP* false positives, *FN* false negatives, *TP* true positives

#### Feature importance

**Fig. 3:**
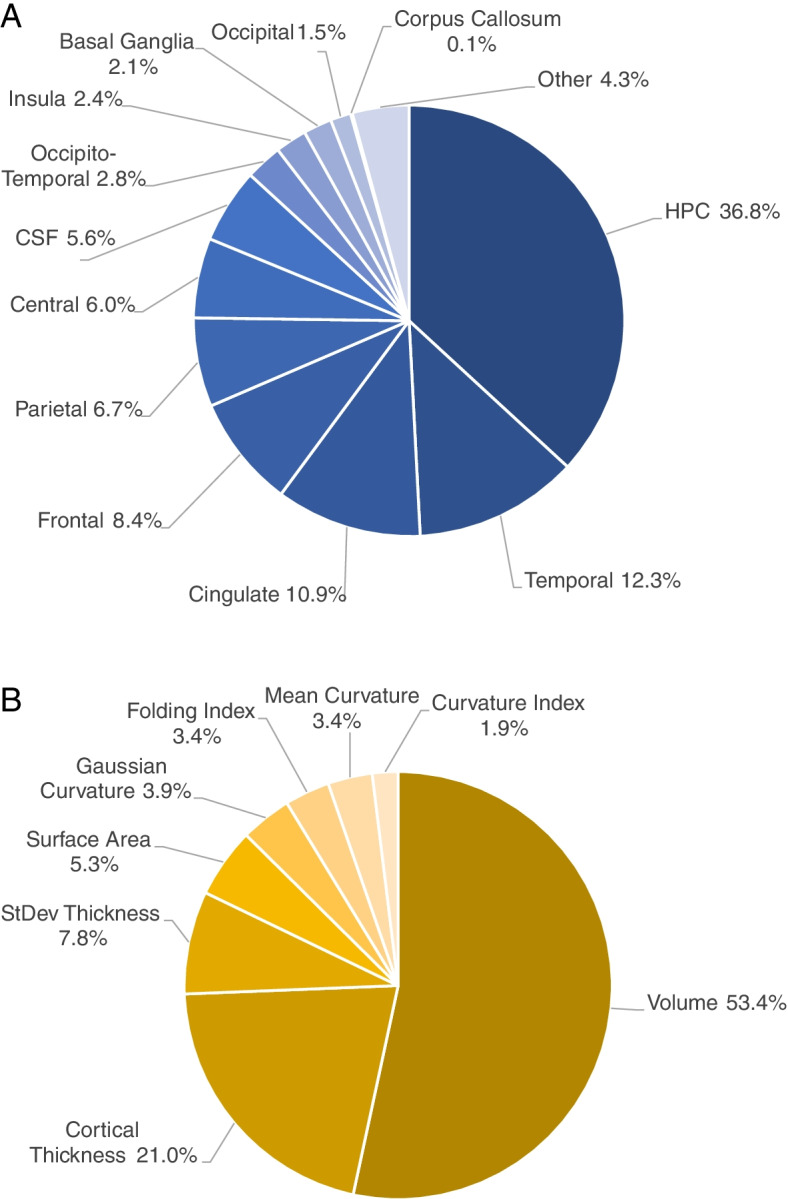
Relative contribution of features for the “HC vs. AD” classifier from experiment A5. Relative contributions are grouped by anatomical region (**A**) and by morphometric feature type (**B**). CSF, cerebrospinal fluid; HPC, hippocampus; StDev Thickness, standard deviation of the cortical thickness

The relative contribution of features to the “HC vs. AD” classifier using ADNI MPRAGE and IR-SPGR for both training and testing (experiment A5) is illustrated grouped by anatomical region (Fig. 3A) and by morphometric feature type (Fig. 3B). Anatomical regions are grouped according to Supplementary Information 2 and detailed contributions for each morphometric feature type-region pair are reported in Supplementary Table 3. We report this experiment as it was the most inclusive, using both scan types in training and testing, and we chose the “HC vs. AD” because it was the best performing binary, giving us confidence that the feature importances are clinically meaningful. Despite, it is important to note that the degree of predictive of features does not necessarily reflect the degree of biological contribution to the disease [49]. We report the classifier without GT features as no improvement was obtained from including those features.

#### Comparison between stable and transitioning diagnoses

Predictions concerning disease progression by the multi-diagnostic classifier (“AD vs MCI vs HC”) from experiment A5 are reported in Table 4. In the test set for that classifier, 38 subjects out of 164 changed diagnosis between the time of scanning and the most recent visit. Considering stable and transition cases separately, our classifier’s performance was similar (*p* = 0.505, i.e., when considering only stable cases, 0.420 MCC, CI 95%: 0.286–0.543; and when only transition cases, 0.327 MCC, CI 95%: 0.071–0.579).

**Table 4:** Classifier confusion matrix for the multi-diagnostic classifier from Experiment A5.

	Classifier prediction		
Ground-truth (2-year)	HC	MCI	AD
**HC**			
HC stable	36	12	4
MCI transition to HC	4	4	0
**MCI**			
MCI stable	29	13	10
HC transition to MCI	2	1	3
**AD**			
AD stable	0	5	18
HC transition to AD	0	0	1
MCI transition to AD	4	7	19

#### Clinical applicability potential

Using our own biomarker evaluation framework published in 2014 [32], we evaluated the “HC vs. MCI vs. AD” classifier (experiment A5) as a biomarker with a “Quality of Evidence” grade of 2 (out of 4), given it is a controlled study with a biomarker defined a priori, based on previous evidence regarding structural MRI alterations in MCI and AD. In terms of “Effect Size,” we converted the AUC metric (77.7% CI 95%: 77.2–82.6%) into odds ratio (OR) [50], resulting in a large OR of 7.06 (CI 95%: 6.75–11.1), which corresponds to a grade 4 (out of 4). The overall sum score of 6 (out of 8) indicates this classifier has potential for clinical applicability and thus should be further evaluated in future randomized controlled trials (RCTs) to compare it to current practices and clinical prediction, in order to determine its real-world clinical usefulness.

### OASIS vs. ADNI

#### Classifier performance

Results from all ADNI vs. OASIS diagnostic experiments using only morphometric features are presented in Table 5. (Results from the corresponding classifiers which received both morphometric and GT features as inputs can be found in the Supplementary Table 2.) All classifiers that received GT features as input alongside morphometric features (experiments B6–B10) made use of GT features for classification. However, inputting GT features to the classifiers had no statistically significant impact on their performance in any experiment (*p* = 0.265–0.998). The classifier distinguishing between ADNI MPRAGE and OASIS scans only selected morphometric features and obtained a BAC of 83.6% [CI 95%: 76.2–90.4%] and MCC of 0.680 [CI 95%: 0.541–0.806] — demonstrating that the datasets are not perfectly distinguishable. For the most inclusive biomarker without GT features (experiment B5), we looked at the data stratified by sex. In the test set, there were 64 females and 63 males. Performance for females was 0.749 MCC [CI 95%: 0.557–0.904] and for males 0.875 MCC [CI 95%: 0.747–0.969], and the difference between sexes was not significant (*p* = 0.219). For females, the classifier output consisted of 30 true negatives (TN), 4 false positives (FP), 4 false negatives (FN), and 26 true positives (TP) while for males the classifier output consisted of 31 TN, 3 FP, 1 FN, and 28 TP.

**Table 5:** Classifier performance for “HC vs. AD” using morphometric features only using ADNI and OASIS subjects

Experiment	Training set	Testing set	MCC [CI: 95%]	BAC [CI: 95%]	ROC AUC [CI: 95%]	Sens [CI: 95%]	Spec [CI: 95%]	PPV (prevalence) [CI: 95%]	NPV (prevalence) [CI: 95%]	PPV (standard) [CI: 95%]	NPV (standard) [CI: 95%]	TN	FP	FN	TP	MCC *p*-value (vs. w/ GT)
B1	ADNI MPRAGE (*N*=194)	ADNI MPRAGE (*N*=84)	0.814 [0.679;0.927]	90.8% [84.1%;96.2%]	97.3% [93.6%;99.6%]	94.9% [87.2%;100.0%]	86.7% [76.2%;95.5%]	81.7% [69.6%;93.3%]	96.4% [90.5%;100.0%]	87.7% [78.6%;95.7%]	94.4% [85.6%;100.0%]	39	6	2	37	0.974
B2	OASIS (*N*=365)	OASIS (*N*=152)	0.564 [0.344;0.771]	75.6% [65.3%;87.2%]	94.1% [89.7%;97.8%]	55.0% [33.3%;77.3%]	96.2% [92.5%;99.2%]	90.1% [73.5%;98.4%]	77.3% [68.9%;87.5%]	93.5% [81.6%;99.0%]	68.1% [58.1%;81.4%]	127	5	9	11	0.998
B3	OASIS (*N*=517)	ADNI MPRAGE (*N*=278)	0.722 [0.657;0.793]	84.3% [80.3%;88.4%]	95.5% [93.1%;97.5%]	70.5% [63.0%;78.2%]	98.0% [95.7%;100.0%]	95.7% [90.2%;100.0%]	84.1% [80.5%;88.0%]	97.2% [93.6%;100.0%]	76.9% [72.1%;82.1%]	146	3	38	91	0.623
B4	ADNI MPRAGE (*N*=278)	OASIS (*N*=517)	0.641 [0.551;0.733]	87.2% [82.4%;97.0%]	94.3% [91.3%;97.0%]	83.6% [74.6%;91.7%]	90.9% [88.3%;93.5%]	85.2% [80.0%;89.8%]	89.9% [84.7%;94.7%]	90.2% [86.4%;93.4%]	84.7% [77.7%;91.8%]	409	41	11	56	0.265
**B5**	**ADNI MPRAGE** **and** **OASIS** **(*N*=295)**	**ADNI MPRAGE** **and** **OASIS** **(*N*=127)**	**0.811** **[0.701;0.906]**	**90.6%** **[85.3%;95.5%]**	**97.4%** **[94.9%;99.3%]**	**91.5%** **[83.6%;98.3%]**	**89.7%** **[81.8%;96.2%]**	**84.8%** **[74.2%;94.2%]**	**94.4%** **[88.9%;98.9%]**	**89.9%** **[82.1%;96.3%]**	**91.4%** **[83.3%;98.3%]**	**61**	**7**	**5**	**54**	**0.383**

MCC *p*-value refers to the *p*-value for the MCC metric for the comparison with the equivalent classifier (i.e., same training and test sets) with morphometric and GT features. PPV/NPV “prevalence” are calculated with an AD prevalence of 38.5% (this corresponds to the prevalence of AD relative to HC based on prevalence estimates from the first visit in the clinical setting of 42.0% for HC and 26.3% for AD) [48]. PPV/NPV “standard” are calculated with a prevalence of 50% to allow for comparison with other studies

*CI* confidence interval, *MCC* Matthew’s correlation coefficient, *ROC AUC* area under the receiver operating characteristic curve, *BAC* balanced accuracy, *Sens* sensitivity, *Spec* specificity, *PPV* positive predict value, *NPV* negative predictive value, *TN* true negatives, *FP* false positives, *FN* false negatives, *TP* true positives. The most global classifier is highlighted

#### Feature importance

**Fig. 4:**
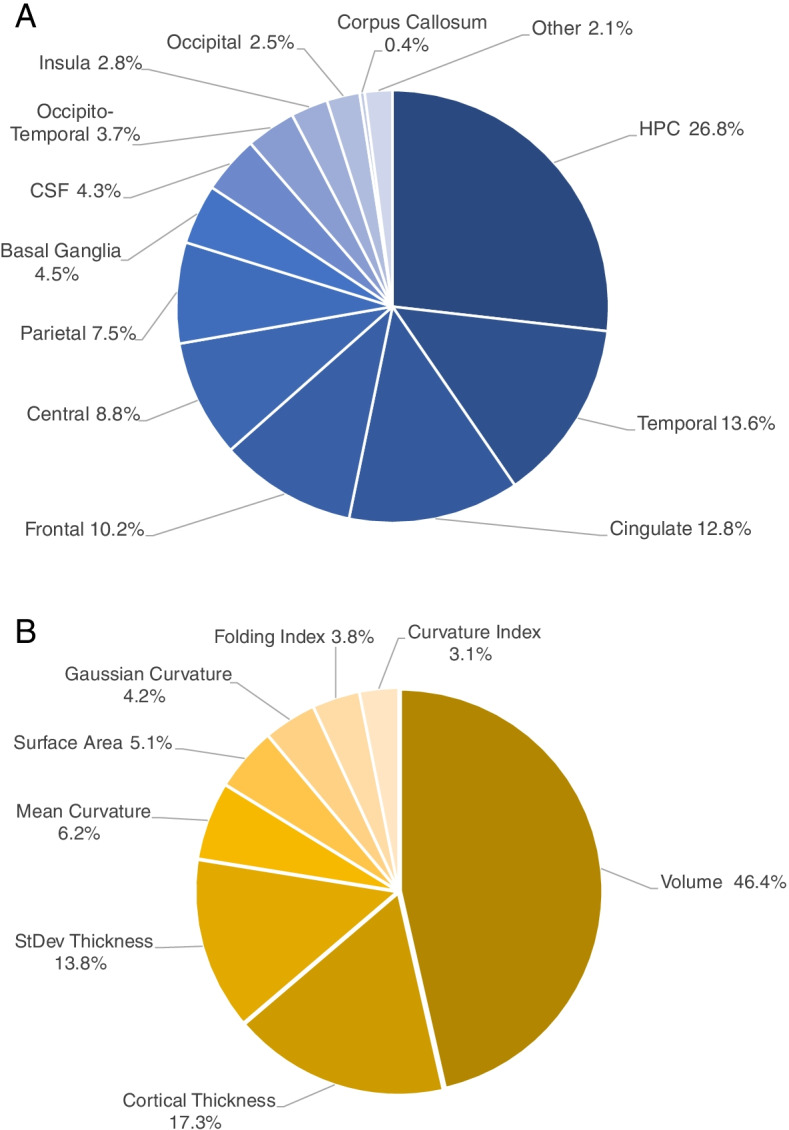
Relative contribution of features for the “HC vs. AD” classifier from experiment B5. Relative contributions are grouped by anatomical region (**A**) and by morphometric feature type (**B**). CSF, cerebrospinal fluid; HPC, hippocampus; StDev Thickness, standard deviation of the cortical thickness

The relative contribution of features for the “HC vs. AD” classifier using ADNI MPRAGE and OASIS subjects for both training and testing (experiment B5) are illustrated grouped by anatomical region (Fig. 4A) and by morphometric feature type (Fig. 4B). Features are grouped by region according to Supplementary Information 2 and detailed contributions for each type-region pair are reported in Supplementary Table 4. We report this experiment as it was the most inclusive, using both datasets in training and testing, and without GT features (as no improvement was obtained from including those features).

### Clinical applicability

Using our own biomarker evaluation framework published in 2014 [32], we evaluated the classifier from experiment B5 as a biomarker with a “Quality of Evidence” grade of 2 (out of 4), given it is a controlled study with a biomarker defined a priori, based on previous evidence regarding structural MRI alterations in MCI and AD. In terms of “Effect Size,” we converted the AUC metric (97.4% CI 95%: 94.9–99.3%) into odds ratio (OR) [50], resulting in a large OR of 146 (CI 95%: 66.4–547), which corresponds to a grade 4 (out of 4). The overall sum score of 6 (out of 8) indicates this classifier has potential for clinical applicability and thus should be further evaluated in future RCT to compare it to current practices and clinical prediction, in order to determine its real-world clinical usefulness.

## Discussion

Early diagnosis of MCI and AD is essential for early therapies and better disease management [6]. For this, we developed a ML-based tool which is (1) multi-diagnostic; (2) trained and tested across 2 independent data sources with multiple acquisition protocols; (3) with tests of generalizability across datasets and protocols, the latter being completely novel in the context of AD (4) based on baseline scans and a follow-up diagnosis, regardless of progression; (5) with transparently reported performance; and (6) with an evaluation of potential clinical applicability. Results showed our tool was well-performing in differentiating AD, MCI, and HC (0.438 MCC; 62.1% BAC; 77.7% AUC), showing an accuracy above chance level (i.e., 33.3% BAC given the 3 possible diagnoses), regardless of diagnosis at the time of scanning, when tested against a follow-up diagnosis of at least 1 year and a maximum of 3 years. Our tool also performed equally well when being tested on data from datasets and acquisition protocols not used in training, meaning it is likely to generalize well to independent data. Additionally, since our classifiers perform equally well without GT features, which add complexity to the classifiers, we found no advantage in including GT features. Finally, we reported the relative importance of different brain regions and morphometric feature type for the diagnostic classification, which might aid in clinical interpretability of the classifiers.

### A MRI-based ML classifier: performance comparison with previous reports

Several recent studies claim classifier interpretability or report feature weights for the classifiers. Those which developed classifiers for AD diagnosis (“HC vs. AD”) obtained accuracies of 85.0% [51] and 87.2% [52], similar to ours of 90.6% BAC on the combined ADNI+OASIS dataset (experiment B5); MCC of 0.666 [53], lower than our ours of 0.811 MCC (experiment B5); and AUC of 98.0% [54], 95.1% [55], and 90.6% [56], comparable to ours of 97.4% (experiment B5). Importantly, all these studies, except [52] and [56], report feature importances at the level of the individual, which we do not. Only one study [53] used an additional AD dataset besides ADNI. Another study [57] using simple ML classifiers and both the ADNI and OASIS obtained a performance similar to ours of 87% BAC on the ADNI dataset, but a lower performance of 70% on the OASIS dataset. While feature weights where not reported, the authors mention having extracted them from the classifiers. Finally, a study [58] reporting landmarks with high discriminative power, when using ADNI-1 for classifier training, obtained an MCC of 0.819 testing in ADNI-2 and 0.839 testing in MIRIAD, which is better than both our experiments where test data was from a different dataset than training data (experiment B8, trained on OASIS and tested on ADNI, had an MCC of 0.739; experiment B9, trained on ADNI and tested on OASIS, had an MCC of 0.674). This study, however, might have suffered from data leakage in the form of biased transfer learning [59].

Our achieved performance on the ADNI dataset alone for the multi-diagnostic “HC vs. MCI vs. AD” (experiment A5: 62.1% BAC, whereas a random classifier would have a BAC of 33%) is comparable to that of recent studies which also report feature weights for the classifiers or claim classifier interpretability. These range from 51.9 to 71.2% in accuracy [54, 60–62]. One study [54] achieved a higher multi-diagnostic “HC vs. MCI vs. AD” accuracy (albeit unbalanced) of 71.2% using a region abnormality score obtained using a deep neural network which, despite providing individual-level abnormality scores, does not make evident how the characteristics of the scan contribute to a region’s abnormality score. Furthermore, this study only considered baseline diagnosis for this classifier, with MCI subjects who had transitioned to AD being labeled as MCI. A second study [60] obtained a “HC vs. MCI vs. AD” multi-diagnostic BAC similar to ours of 62.5% on the ADNI dataset using an ensemble of random forest classifiers, which is highly interpretable. A third study [61] reporting feature importances from a random forest classifier obtained an “HC vs. MCI vs. AD” multi-diagnostic BAC of 51.9%, which was lower than ours. A fourth study [62] obtained a similar (vs. ours) “HC vs. MCI vs. AD” multi-diagnostic BAC of 61.9% using an ensemble of linear SVMs, but do not report feature importances, only the frequency with which each feature was selected by the ensemble. Finally, the latter three studies only considered MCI-to-AD transitions when labeling subjects [60–62].

### Hippocampal and cingulate, frontal, and other temporal changes contribute most

Extracted feature contributions by brain regions obtained from the “HC vs. AD” classifiers (experiments A5 and B5, in Figs. 3A and 4A, respectively) are in accordance with our current etiological and neuroscientific understanding of AD. Hippocampal features were the strongest contributors to the classification, at approximately 25–45%, which is consistent with the understanding that hippocampal atrophy is a structural hallmark of AD in the brain [63]. Temporal regions were the next most highly weighted, at approximately 13%, followed by cingulate and frontal regions, each contributing approximately 8–12% for the classifier decision, and all have been independently associated with AD and its progression [64–66]. The remaining regions combined contribute approximately 25–40%, and all regions make meaningful contributions to the decision, speaking to the fact that AD is a disease with brain-wide effects.

### Volume changes contribute most

Extracted feature contributions by feature type obtained from the same “HC vs. AD” classifiers as above (experiments A5 and B5, in Figs. 3B and 4B, respectively) reveal that volumes are the strongest contributors to the classification, at approximately 45–55%, followed by cortical thicknesses, at approximately 17–21%, which is in line with evidence of brain atrophy [39] and cortical thinning in AD [40]. The combination of gyrification measures (mean curvature, Gaussian curvature, folding index, and curvature index) contributed 12–17% to the decision, which is consistent with previous evidence associating cortical gyrification with AD [41]. While we did not investigate the performance gained from introducing different types of measures, we show that all types of measures used had an important contribution to the classification decision. Furthermore, care should be taken not to interpret these contributions outside of the classification context, as the predictive value of features does not necessarily reflect biological contribution to the disease [49].

### MRI morphometric-based graph theory complexity may be unnecessary

When introducing complex features, such as GT-based ones, it is important to determine whether this brings an improvement, as complexity sacrifices clinical interpretability in ML. In fact, we did not observe a statistically significant improvement when GT features were used as input along with morphometric features (*p* = 0.060–0.998). However, the fact that both the classifier built to distinguish protocols (“ADNI MPRAGE vs. ADNI IR-SPGR”) and the classifier built to distinguish datasets (“OASIS vs. ADNI”) did not “choose” to use GT-based metrics even when these were given as input, this indicates that GT metrics may be more robust to protocol and dataset differences, which could have implications in classifier generalizability. It is important to note, as a limitation, that since models were trained exclusively on either morphometric or graph theory data, complementarity of these data might have been lost. The same is true for complementarity across GT binarization thresholds. This loss of complementarity could be particularly relevant when using larger datasets and more complex models. Altogether, the fact that no improvement is obtained from inputting GT metrics suggests that these may be unnecessary when small sample sizes and simple models are used and that the benefit of their inclusion (which might be observed with larger samples and more complex algorithm) should be evaluated as they introduce additional complexity in the models.

### Generalizability across the two most common acquisition protocols

Comparing the results of experiments A1 and A2 with those of experiment A5 from Table 2 shows that combining IR-SPGR and MPRAGE scans in training and testing results in similar performance when compared to building classifiers on a single scanning protocol (“HC vs. MCI vs. AD”: 0.350 MCC for MPRAGE-only (*p* = 0.285) and 0.263 MCC for IR-SPGR-only (*p* = 0.160) vs. 0.438 MCC for the combination). Importantly, the considerably larger training set for the combination classifier (*n* = 379 for the combination vs. *n* = 106 for the IR-SPGR-only classifier and *n* = 295 for the MPRAGE-only classifier) did not result in any improvement, suggesting that the disease effects are fairly large and can be detected with small sample sizes using simple ML classifiers and thus that more complex models for AD diagnostic may be unnecessary.

Moreover, the results from experiments A3 and A4 demonstrate that despite the protocols being almost perfectly distinguishable by the classifiers, a classifier trained on one protocol can classify scans of another protocol with similar performance as a classifier trained on that other protocol (“HC vs. MCI vs. AD”: 0.350 MCC on experiment A1 vs. 0.372 MCC on experiment A4 (*p* = 0.766); 0.262 MCC on experiment A2 vs. 0.424 MCC on experiment A3 (*p* = 0.207)). This similarity in performance shows that a biomarker using our features and algorithm, although trained in one dataset, is well adapted to classify patients whose scan is acquired using other MRI acquisition protocols. This is the case because the underlying physiopathology dominates in the algorithm’s decision as opposed to eventual sequence differences or image signal differences. Finally, we demonstrated that MPRAGE and IR-SPGR protocols are almost perfectly distinguishable using only morphometric features (0.968 MCC; 99.2% BAC) — ascertaining that these protocols should not be merged without balancing them across diagnoses, as otherwise a classifier might be biased if it learns to use disease-irrelevant protocol-related differences in features to attribute labels.

### Generalizability across independent patient datasets

Similarly to what was observed in the acquisition protocol comparison, comparing the results of experiments B1 and B2 with those of experiment B5 from Table 5 shows that combining ADNI MPRAGE and OASIS MPRAGE scans in training and testing can significantly increase performance when compared to building classifiers on a single dataset (“HC vs. AD”: 0.814 MCC for ADNI-only (*p* = 0.955) and 0.564 MCC for OASIS-only (*p* = 0.028) vs. 0.811 MCC for the combination). These results demonstrate that combining data from different sources can be preferential to training a classifier for each data source, as not only performance can significantly increase, but also a more robust classifier is being trained, as it can classify subjects from multiple sources. Importantly, the OASIS-only classifier from experiment B2 had a larger training sample than the dataset combination classifier from experiment B5 (*n* = 365 and *n* = 295, respectively), meaning that the increase in performance observed cannot be attributed to a larger training set. Furthermore, when showing that ADNI and OASIS datasets are not perfectly distinguishable (MCC = 0.680; 83.6% BAC), we consequently also show that that applies to the MPRAGE scans with a 2-factor acceleration (which were used in OASIS) vs. those with non-accelerated MPRAGE scans (used in ADNI). This contrasts with the “MPRAGE vs. IR-SPGR” distinction (0.968 MCC; 99.2% BAC), which was nearly perfect, and is consistent with previous evidence showing that acceleration has little impact on brain atrophy metrics, with effects being dominated by MPRAGE vs. IR-SPGR differences [15]. Finally, results from experiments B3 and B4 demonstrate that a classifier trained on one dataset can classify scans of another dataset with similar performance as a classifier trained on that other dataset (“HC vs. AD”: 0.814 MCC on experiment B1 vs. 0.722 MCC on experiment B3 (*p* = 0.211); 0.564 MCC on experiment B2 vs. 0.641 MCC on experiment B4 (*p* = 0.461)). These results are in line with previous evidence showing that a classifier trained on ADNI data can be used to classify OASIS data with similar performance as a classifier trained on OASIS data [57], although we obtained a slightly higher performance of 87.2% BAC compared to 75.6% BAC when training with ADNI and testing with OASIS.

### Sex-stratified results

Sex-stratified data showed no difference in performance between males and females within the ADNI dataset for the multi-diagnostic classifier (experiment A5) (0.424 MCC females, 0.454 MCC for males, 0. MCC overall, *p* = 0.438), nor in the combined ADNI and OASIS datasets for the “HC vs. AD” task (experiment B5) (0.875 MCC females, 0.749 MCC for males, 0.811 MCC overall, *p* = 0.219). Despite no differences being observed in our biomarkers, reporting sex-stratified data remains important as differences in performance between sexes have implications on the potential translation of the classifiers into clinical practice. Balancing testing sets for sex is essential to ensure that sex proportions in the training set do not inflate test results, especially when there is a significant difference between diagnoses in sex, such as in our datasets. While sex proportions could be adjusted in the training set, this could mean reducing the size of the test set, and also it would not allow for reporting of sex-stratified results with the same confidence for both sexes.

### Performance in stable and transitioning diagnoses

Our classifier from experiment A5 performed equally well with stable (0.420 MCC) and transition cases (0.327 MCC), which suggest that imaging changes might precede diagnostic changes. Importantly, the classifier performed poorly on the HC to MCI progression and MCI to HC regression transitions. Given the small number of these transitions in the test set (7 MCI-to-HC and 6 HC-to-MCI), their estimated performance has a wide error margin. Nonetheless, the poor performance estimated for these cases demonstrates that excluding transitions we cannot reliably predict may artificially inflate model performance. This is due to the fact that when using the classifier as intended, there is no way to exclude these transitions, as the future state of the patient is unknown (which is exactly what the classifier is being used to predict). Therefore, it is important to train and test the classifier with a population that best resembles the intended use of the classifier (i.e., including all possible transition cases) even if including these transitions reduces classifier performance. Furthermore, while many studies differentiate stable from progressing MCI [14], failure to account for other transitions reduces their potential applicability, as predicting a progression from MCI to AD is not necessarily more useful than predicting a progression from HC to MCI, or a regression from MCI to HC. For example, accurately predicting future MCI in currently HC patients indicates that their brain already shows some pattern of structural change before symptom onset. Early intervention in these cases (recommending mentally stimulating activities, monitoring the appearance of symptoms, etc.) could result in better outcomes and delayed onset of symptoms. Similarly, predicting regression from MCI to HC could be a good indicator that the current clinical intervention and therapies are working.

### Clinical applicability and usefulness

Both the classifiers we evaluated obtained a clinical applicability score of 6 (“HC vs. MCI vs. AD” from experiment A5 and “HC vs. AD” from experiment B5). Despite the better performance of the second classifier, we consider that the multi-class has more potential to be clinically useful, as it more closely approximates the decision the clinicians face. Given this evidence, we consider that the present MRI biomarker should undergo evaluation in future RCT to compare it to current practices in terms of real-world clinical usefulness, which should include measures of risk, convenience, cost, number-needed-to–assess and outcome relevance, at least [32], as it may have potential to improve clinicians’ performance as a first-line biomarker — before invasive, less available, and more expensive ones such as PET or CSF readings are used — at an initial stage.

### Limitations and future work

The present study was limited by the fact that only one of the data sources included patients with MCI. Furthermore, the testing set is not identical to the clinical situation faced by physicians in the clinical decision process. This or any future classifier built using the proposed approach on a larger, longitudinal, multi-site dataset would have to be thoroughly evaluated in the clinical setting before it can be adopted. As such, as mentioned in the previous section, future work should assess the clinicians’ performance with and without the information from the algorithm so its potential impact on patients can be understood. Also, our classifiers did not make use of cognitive features or scores, which could be of high predictive value and can be easily acquired. Finally, while we provide some insight into the classifier decision with the goal of improving interpretability, whether classifier outputs would be correctly interpreted by clinicians without a good understanding of the statistical rationale behind ML algorithms remains to be verified.

## Conclusions

In sum, our approach allowed us to develop ML classifiers with high performance in the distinction between HC and patients with AD, as well as with reasonable performance in the multi-diagnostic distinction of HC, MCI, and AD subjects. Importantly, these classifiers are robust to transition cases and identify some future AD cases earlier than clinicians. Our approach of using an ensemble of classifiers using linear or tree-based algorithms allowed for the development of a complex classifier which outputs the importance that each brain region had on the classifiers’ predictions, which may allow for easier interpretation by clinicians. It also outputs a probability of a diagnosis which allows clinicians to consider the confidence of the classifier when evaluating its output. Our work also demonstrated the possibility of combining different MRI protocols and datasets in the training of these algorithms and opens the road for the future development of algorithms using a similar strategy and features with larger and more diverse datasets. Given the performance of our most inclusive multi-diagnostic classifiers, they may be clinically useful provided they are successful in future prospective RCT controlled studies [32].

## Supplementary Information

**Additional file 1.** Supplementary Information for “Early diagnosis of Alzheimer’s disease using machine learning: a multi-diagnostic, generalizable approach”.

## Data Availability

All ADNI data presented is available through the ADNI website at http://adni.loni.usc.edu/data-samples/access-data/. All OASIS data is available through the OASIS website at https://www.oasis-brains.org/.

## Abbreviations

AD: Alzheimer’s disease
ADNI: Alzheimer’s Disease Neuroimaging Initiative
BAC: Balanced accuracy
CDR: Clinical Dementia Rating
CI: Confidence interval
CSF: Cerebrospinal fluid
CV: Cross-validation
DT: Decision tree
ET: Extremely randomized tree
FN: False negatives
FP: False positives
GT: Graph theory
HC: Healthy controls
IR-SPGR: Spoiled gradient echo with inversion recovery preparation
l-SVM: Linear support vector machine
LDA: Linear discriminant analysis
LR: Logistic regression
LR-SGD: Logistic regression classifier with stochastic gradient descent learning
MCC: Matthew’s correlation coefficient
MCI: Mild cognitive impairment
ML: Machine learning
MMSE: Mini-Mental State Examination
MPRAGE: Magnetization-prepared rapid gradient echo
MRI: Magnetic resonance imaging
MTA: Medial temporal atrophy
NPV: Negative predictive value
OASIS: Open Access Series of Imaging Studies
OD: Odds ratio
PET: Positron emission tomography
PPV: Positive predict value
RF: Random forest
RCT: Randomized controlled trial
AUC: Area under the receiver operating characteristic curve
ROI: Region of interest
Sens: Sensitivity
Spec: Specificity
TN: True negatives
TP: True positives

## References

[1] Prince M, Wimo A, Guerchet M, Ali G-C, Yu-Tzu W, Prina M (2015). World Alzheimer Report 2015: The Global Impact of Dementia: an analysis of prevalence, incidence, cost and trends.

[2] Plassman BL, Langa KM, Fisher GG, Heeringa SG, Weir DR, Ofstedal MB (2007). Prevalence of dementia in the United States: the aging, demographics, and memory study. Neuroepidemiology..

[3] Dubois B, Feldman HH, Jacova C, Hampel H, Molinuevo JL, Blennow K (2014). Advancing research diagnostic criteria for Alzheimer’s disease: the IWG-2 criteria. Lancet Neurol.

[4] Albert MS, DeKosky ST, Dickson D, Dubois B, Feldman HH, Fox NC (2011). The diagnosis of mild cognitive impairment due to Alzheimer’s disease: recommendations from the National Institute on Aging-Alzheimer’s Association workgroups on diagnostic guidelines for Alzheimer’s disease. Alzheimers Dement.

[5] Misra C, Fan Y, Davatzikos C (2009). Baseline and longitudinal patterns of brain atrophy in MCI patients, and their use in prediction of short-term conversion to AD: results from ADNI☆. NeuroImage..

[6] Crous-Bou M, Minguillón C, Gramunt N, Molinuevo JL. Alzheimer’s disease prevention: from risk factors to early intervention. Alzheimers Res Ther. 2017;9(1) [cited 2019 Dec 2]. Available from: http://alzres.biomedcentral.com/articles/10.1186/s13195-017-0297-z. 10.1186/s13195-017-0297-z PMC5596480 28899416

[7] Bocchetta M, Galluzzi S, Kehoe PG, Aguera E, Bernabei R, Bullock R (2015). The use of biomarkers for the etiologic diagnosis of MCI in Europe: an EADC survey. Alzheimers Dement.

[8] Khoury R, Ghossoub E (2019). Diagnostic biomarkers of Alzheimer’s disease: a state-of-the-art review. Biomark Neuropsychiatry.

[9] Jack CR, Knopman DS, Jagust WJ, Shaw LM, Aisen PS, Weiner MW (2010). Hypothetical model of dynamic biomarkers of the Alzheimer’s pathological cascade. Lancet Neurol.

[10] Pellegrini E, Ballerini L, Hernandez M d CV, Chappell FM, González-Castro V, Anblagan D (2018). Machine learning of neuroimaging for assisted diagnosis of cognitive impairment and dementia: a systematic review. Alzheimers Dement Diagn Assess Dis Monit.

[11] Tanveer M, Richhariya B, Khan RU, Rashid AH, Khanna P, Prasad M (2020). Machine learning techniques for the diagnosis of Alzheimer’s disease: a review. ACM Trans Multimed Comput Commun Appl.

[12] Koepsell TD, Monsell SE (2012). Reversion from mild cognitive impairment to normal or near-normal cognition: risk factors and prognosis. Neurology..

[13] Ranginwala NA, Hynan LS, Weiner MF, White CL (2008). Clinical criteria for the diagnosis of Alzheimer disease: still good after all these years. Am J Geriatr Psychiatry.

[14] Ansart M, Epelbaum S, Bassignana G, Bône A, Bottani S, Cattai T, et al. Predicting the progression of mild cognitive impairment using machine learning: A systematic, quantitative and critical review. Medical Image Analysis. 2021;67:101848. 10.1016/j.media.2020.101848 33091740

[15] Leung KK, Malone IM, Ourselin S, Gunter JL, Bernstein MA, Thompson PM (2015). Effects of changing from non-accelerated to accelerated MRI for follow-up in brain atrophy measurement. NeuroImage..

[16] Lin C, Watson RE, Ward HA, Rydberg CH, Witte RJ, Bernstein MA (2006). MP-RAGE compared to 3D IR SPGR for optimal T1 contrast and image quality in the brain at 3T. Proc Intl Soc Mag Reson Med.

[17] Castelvecchi D (2016). Can we open the black box of AI?. Nature..

[18] Tong T, Gray K, Gao Q, Chen L, Rueckert D (2017). Multi-modal classification of Alzheimer’s disease using nonlinear graph fusion. Pattern Recognit.

[19] Raamana PR, Strother SC (2020). for the Australian Imaging Biomarkers, Lifestyle flagship study of ageing, for The Alzheimer’s Disease Neuroimaging Initiative. Does size matter? The relationship between predictive power of single-subject morphometric networks to spatial scale and edge weight. Brain Struct Funct.

[20] Hett K, Ta V-T, Oguz I, Manjón JV, Coupé P (2021). Multi-scale graph-based grading for Alzheimer’s disease prediction. Med Image Anal.

[21] Raamana PR, Weiner MW, Wang L, Beg MF (2015). Thickness network features for prognostic applications in dementia. Neurobiol Aging.

[22] He Y, Chen Z, Evans A (2008). Structural insights into aberrant topological patterns of large-scale cortical networks in Alzheimer’s disease. J Neurosci.

[23] Pereira JB, Mijalkov M, Kakaei E, Mecocci P, Vellas B, Tsolaki M (2016). Disrupted network topology in patients with stable and progressive mild cognitive impairment and Alzheimer’s disease. Cereb Cortex.

[24] Tijms BM, ten Kate M, Gouw AA, Borta A, Verfaillie S, Teunissen CE (2018). Gray matter networks and clinical progression in subjects with predementia Alzheimer’s disease. Neurobiol Aging.

[25] Katiyar S, Rani TS, Bhavani SD, Goswami D, Hoang TA (2021). Exploring Alzheimer’s disease network using social network analysis. Distributed computing and Internet technology.

[26] Phillips DJ, McGlaughlin A, Ruth D, Jager LR, Soldan A (2015). Graph theoretic analysis of structural connectivity across the spectrum of Alzheimer’s disease: the importance of graph creation methods. NeuroImage Clin.

[27] Mårtensson G, Pereira JB, Mecocci P, Vellas B, Tsolaki M, Kłoszewska I (2018). Stability of graph theoretical measures in structural brain networks in Alzheimer’s disease. Sci Rep.

[28] Scarpazza C, Ha M, Baecker L, Garcia-Dias R, Pinaya WHL, Vieira S (2020). Translating research findings into clinical practice: a systematic and critical review of neuroimaging-based clinical tools for brain disorders. Transl Psychiatry.

[29] Delgado R, Tibau X-A (2019). Why Cohen’s Kappa should be avoided as performance measure in classification. Gu Q, editor. PLoS One.

[30] Dinga R, Penninx BWJH, Veltman DJ, Schmaal L, Marquand AF. Beyond accuracy: measures for assessing machine learning models, pitfalls and guidelines. bioRxiv. 2019; [cited 2019 Dec 2]; Available from: http://biorxiv.org/lookup/doi/10.1101/743138.

[31] Heston TF (2011). Standardizing predictive values in diagnostic imaging research. J Magn Reson Imaging.

[32] Prata D, Mechelli A, Kapur S (2014). Clinically meaningful biomarkers for psychosis: a systematic and quantitative review. Neurosci Biobehav Rev.

[33] Ruigrok ANV, Salimi-Khorshidi G, Lai M-C, Baron-Cohen S, Lombardo MV, Tait RJ (2014). A meta-analysis of sex differences in human brain structure. Neurosci Biobehav Rev.

[34] Lockhart SN, DeCarli C (2014). Structural imaging measures of brain aging. Neuropsychol Rev.

[35] LaMontagne PJ, Benzinger TLS, Morris JC, Keefe S, Hornbeck R, Xiong C, et al. OASIS-3: longitudinal neuroimaging, clinical, and cognitive dataset for normal aging and Alzheimer disease. Radiol Imaging. 2019; [cited 2020 Aug 17]. Available from: http://medrxiv.org/lookup/doi/10.1101/2019.12.13.19014902.

[36] Wonderlick J, Ziegler D, Hosseinivarnamkhasti P, Locascio J, Bakkour A, Vanderkouwe A (2009). Reliability of MRI-derived cortical and subcortical morphometric measures: effects of pulse sequence, voxel geometry, and parallel imaging. NeuroImage..

[37] Vemuri P, Senjem ML, Gunter JL, Lundt ES, Tosakulwong N, Weigand SD (2015). Accelerated vs. unaccelerated serial MRI based TBM-SyN measurements for clinical trials in Alzheimer’s disease. NeuroImage..

[38] Fischl B, Salat DH, Busa E, Albert M, Dieterich M, Haselgrove C (2002). Whole brain segmentation. Neuron..

[39] Pini L, Pievani M, Bocchetta M, Altomare D, Bosco P, Cavedo E (2016). Brain atrophy in Alzheimer’s disease and aging. Ageing Res Rev.

[40] Singh V, Chertkow H, Lerch JP, Evans AC, Dorr AE, Kabani NJ (2006). Spatial patterns of cortical thinning in mild cognitive impairment and Alzheimer’s disease. Brain..

[41] Liu T, Lipnicki DM, Zhu W, Tao D, Zhang C, Cui Y (2012). Cortical gyrification and sulcal spans in early stage Alzheimer’s disease. Ginsberg SD, editor. PLoS One.

[42] Radanovic M, Pereira FRS, Stella F, Aprahamian I, Ferreira LK, Forlenza OV (2013). White matter abnormalities associated with Alzheimer’s disease and mild cognitive impairment: a critical review of MRI studies. Expert Rev Neurother.

[43] Iglesias JE, Augustinack JC, Nguyen K, Player CM, Player A, Wright M (2015). A computational atlas of the hippocampal formation using ex vivo, ultra-high resolution MRI: Application to adaptive segmentation of in vivo MRI. NeuroImage..

[44] van der Flier WM, Scheltens P (2009). Hippocampal volume loss and Alzheimer disease progression. Nat Rev Neurol.

[45] Rubinov M, Sporns O (2010). Complex network measures of brain connectivity: uses and interpretations. NeuroImage..

[46] Fortin F-A, De Rainville F-M, Gardner M-AG, Parizeau M, Gagné C (2012). DEAP: evolutionary algorithms made easy. J Mach Learn Res.

[47] Altman DG, Bland JM (1994). Statistics notes: diagnostic tests 2: predictive values. BMJ..

[48] Davis M, O’connell T, Johnson S, Cline S, Merikle E, Martenyi F (2018). Estimating Alzheimer’s disease progression rates from normal cognition through mild cognitive impairment and stages of dementia. Curr Alzheimer Res.

[49] Haufe S, Meinecke F, Görgen K, Dähne S, Haynes J-D, Blankertz B (2014). On the interpretation of weight vectors of linear models in multivariate neuroimaging. NeuroImage..

[50] Salgado JF (2018). Transforming the area under the normal curve (AUC) into Cohen’s d, Pearson’s r pb, odds-ratio, and natural log odds-ratio: two conversion tables. Eur J Psychol Appl Leg Context.

[51] Shahamat H, Saniee AM (2020). Brain MRI analysis using a deep learning based evolutionary approach. Neural Netw.

[52] Khedher L, Illán IA, Górriz JM, Ramírez J, Brahim A, Meyer-Baese A (2017). Independent component analysis-support vector machine-based computer-aided diagnosis system for Alzheimer’s with visual support. Int J Neural Syst.

[53] Qiu S, Joshi PS, Miller MI, Xue C, Zhou X, Karjadi C (2020). Development and validation of an interpretable deep learning framework for Alzheimer’s disease classification. Brain..

[54] Lee E, Choi J-S, Kim M, Suk H-I (2019). Toward an interpretable Alzheimer’s disease diagnostic model with regional abnormality representation via deep learning. NeuroImage..

[55] Lian C, Liu M, Zhang J, Shen D (2020). Hierarchical fully convolutional network for joint atrophy localization and Alzheimer’s disease diagnosis using structural MRI. IEEE Trans Pattern Anal Mach Intell.

[56] Liu M, Cheng D, Wang K, Wang Y (2018). the Alzheimer’s Disease Neuroimaging Initiative. Multi-modality cascaded convolutional neural networks for Alzheimer’s disease diagnosis. Neuroinformatics..

[57] Samper-González J (2018). Reproducible evaluation of classification methods in Alzheimer’s disease: Framework and application to MRI and PET data.

[58] Liu M, Zhang J, Adeli E, Shen D (2018). Landmark-based deep multi-instance learning for brain disease diagnosis. Med Image Anal.

[59] Wen J, Thibeau-Sutre E, Diaz-Melo M, Samper-González J, Routier A, Bottani S (2020). Convolutional neural networks for classification of Alzheimer’s disease: overview and reproducible evaluation. Med Image Anal.

[60] Ramírez J, Górriz JM, Ortiz A, Martínez-Murcia FJ, Segovia F, Salas-Gonzalez D (2018). Ensemble of random forests One vs. Rest classifiers for MCI and AD prediction using ANOVA cortical and subcortical feature selection and partial least squares. J Neurosci Methods.

[61] Donnelly-Kehoe PA, Pascariello GO, Gómez JC (2018). Looking for Alzheimer’s disease morphometric signatures using machine learning techniques. J Neurosci Methods.

[62] Sørensen L, Nielsen M (2018). Ensemble support vector machine classification of dementia using structural MRI and mini-mental state examination. J Neurosci Methods.

[63] Maruszak A, Thuret S. Why looking at the whole hippocampus is not enough—a critical role for anteroposterior axis, subfield and activation analyses to enhance predictive value of hippocampal changes for Alzheimer’s disease diagnosis. Front Cell Neurosci. 2014;8 [cited 2020 Sep 15]. Available from: http://journal.frontiersin.org/article/10.3389/fncel.2014.00095/abstract. 10.3389/fncel.2014.00095 PMC3978283 24744700

[64] Ansari MA, Scheff SW (2010). Oxidative stress in the progression of Alzheimer disease in the frontal cortex. J Neuropathol Exp Neurol.

[65] Jack CR, Petersen RC, Xu Y, O’Brien PC, Smith GE, Ivnik RJ (1998). Rate of medial temporal lobe atrophy in typical aging and Alzheimer’s disease. Neurology..

[66] Mutlu J, Landeau B, Tomadesso C, de Flores R, Mézenge F, de La Sayette V, et al. Connectivity disruption, atrophy, and hypometabolism within posterior cingulate networks in Alzheimer’s disease. Front Neurosci. 2016;10 [cited 2020 Oct 29]. Available from: http://journal.frontiersin.org/article/10.3389/fnins.2016.00582/full. 10.3389/fnins.2016.00582 PMC5174151 28066167

