# Supplementary material for: Early diagnosis of Alzheimer’s disease using machine learning: a multi-diagnostic, generalizable approach
Source: Alzheimers Res Ther. 2022 Aug 3;14:107. doi: 10.1186/s13195-022-01047-y (PMC9347083; doi:10.1186/s13195-022-01047-y)
Supplement: Supplementary file 1 — Additional file 1. Supplementary Information for “Early diagnosis of Alzheimer’s disease using machine learning: a multi-diagnostic, generalizable approach”. [file 13195_2022_1047_MOESM1_ESM.docx]

**Supplementary Information for “Early diagnosis of Alzheimer’s disease using machine learning: a multi-diagnostic, generalizable approach”**

Supplementary Information 1 **–** *Hyperparameters tested for each algorithm and possible values*

l-SVM: C (2e-12, 2e-10, 2e-8, 2e-6, 2e-4, 2e-2, 2e0, 2e2, 2e4, 2e6, 2e8, 2e10, 2e12)

DT: Splitter (‘best’, ‘random); Maximum depth (2, 5, 15, ‘None’); Minimum samples for split (1, 2, 5, 15, 40); Minimum samples for leaf (1, 2, 5, 10, 20); Maximum features for split (‘log2’, ‘sqrt’, ‘None)

RF: Number of trees (2, 5, 10, 25, 50); Maximum depth (2, 5, 15, ‘None’); Minimum samples for split (1, 2, 5, 15, 40); Minimum samples for leaf (1, 2, 5, 10, 20); Maximum features for split (‘log2’, ‘sqrt’, ‘None); Maximum leaf nodes (2, 5, 10, ‘None’); Out-of-bag samples (‘True’, ‘False’); Maximum samples (0.3, 0.6, 0.9).

ET: Number of trees (2, 5, 10, 25, 50); Maximum depth (2, 5, 15, ‘None’); Minimum samples for split (1, 2, 5, 15, 40); Minimum samples for leaf (1, 2, 5, 10, 20); Maximum features for split (‘log2’, ‘sqrt’, ‘None); Out-of-bag samples (‘True’, ‘False’); Maximum samples (0.3, 0.6, 0.9).

LDA: Solver (‘svd’, ‘lsqr’)

LR: C (2e-12, 2e-10, 2e-8, 2e-6, 2e-4, 2e-2, 2e0, 2e2, 2e4, 2e6, 2e8, 2e10, 2e12); Penalty (‘l1’, ‘l2’); Solver (‘liblinear’, ‘saga’)

LR-SGD: Penalty (‘l1’, ‘l2’, ‘elasticnet’); Alpha (1e-3; 1e-4, 1e-5; Maximum iterations (100, 500, 1000, 2000); Learning rate exponent (0.25, 0.5, 0.75); Elastic Net mixing parameter (0.15, 0.30, 0.50, 0.70, 0.85) [Only used if Penalty is ‘elasticnet’]

Supplementary Information 2 **-** *Grouping of FreeSurfer anatomical labels to anatomical regions for feature importance grouping*

Basal Ganglia: Accumbens Area; Amygdala; Caudate; Putamen; Pallidum; Thalamus Proper; Ventral Diencephalon.

Central: ParaCentral; PostCentral; PreCentral.

Cingulate: Caudal Anterior Cingulate; Caudal Middle Frontal Cingulate; Isthmus Cingulate; Posterior Cingulate; Rostral Anterior Cingulate; Rostral Middle Frontal Cingulate

Corpus Callosum: Anterior Corpus Callosum; Central Corpus Callosum; Mid Anterior Corpus Callosum; Mid Posterior Corpus Callosum; Posterior Corpus Callosum;

Cerebrospinal Fluid: 3rd Ventricle; 4th Ventricle; Inferior Lateral Ventricles; Lateral Ventricles; Total CSF

Frontal: Frontal Pole; Lateral Orbito-Frontal; Medial Orbito-Frontal; Pars Opercularis; Pars Orbitalis; Pars Triangularis; Superior Frontal

Hippocampus: CA1; CA3; CA4; Entorhinal Cortex; Fimbria; Granule Cell and Molecular Layer of the Dentate Gyrus; Hippocampal Fissure; Hippocampal Tail; Hippocampal-Amygdaloid Transition Region; Molecular Layer; Parasubiculum; Presubiculum; Subiculum; Whole Hippocampus

Insula: Insula

Occipital: Lateral Occipital; Peri Calcarine

Occipito-Temporal: Fusiform Gyrus; Lingual Gyrus

Parietal: Cuneus; Inferior Parietal; Precuneus; Superior Parietal; Supramarginal Gyrus

Temporal: Banks of the Superior Temporal Sulcus; Inferior Temporal; Middle Temporal; Superior Temporal; Temporal Pole; Transverse Temporal

Others: Brain Stem; Cerebellum; Cerebral White Matter Volume; Choroid Plexus; Cortex Volume; Estimated Total Intracranial Volume; Non-WM hypointensities; Optic Chiasm; Subcortical Gray Matter; Surface Holes; Total Gray Matter Volume; Unsegmented White Matter; Vessel; WM hypointensities

Supplementary Table 1 – *Classifier performance using morphometric features and GT features for classifiers using ADNI subjects with IR-SPGR or MPRAGE scans.*

| Experiment | Training Set | Testing Set | Classification task | MCC [CI: 95%] | BAC [CI: 95%] | ROC AUC [CI: 95%] | Sens [CI: 95%] | Spec [CI: 95%] | PPV  (prevalence) [CI: 95%] | NPV (prevalence) [CI: 95%] | PPV (standard) [CI: 95%] | NPV (standard) [CI: 95%] | TN | FP | FN | TP |
| --- | --- | --- | --- | --- | --- | --- | --- | --- | --- | --- | --- | --- | --- | --- | --- | --- |
| A6 | ADNI MPRAGE (N=295) | ADNI MPRAGE (N=128) | HC vs. MCI | 0.260 [0.060;0.454] | 62.8% [52.9%;72.7%] | 66.2% [54.9%;77.0%] | 54.5% [40.0%;68.9%] | 71.1% [57.1%;84.1%] | 45.5% [29.2%;67.5%] | 77.9% [68.3%;85.9%] | 65.3% [48.3%;81.3%] | 61.0% [48.8%;73.0%] | 32 | 13 | 20 | 24 |
|  |  |  | HC vs. AD | 0.810 [0.670;0.929] | 90.6% [84.0%;90.5%] | 97.8% [94.7%;99.7%] | 92.3% [82.9%;100.0%] | 88.9% [78.6%;97.6%] | 83.9% [70.8%;96.3%] | 94.9% [88.0%;100.0%] | 89.3% [79.5%;97.7%] | 92.0% [82.1%;100.0%] | 40 | 5 | 3 | 36 |
|  |  |  | MCI vs. AD | 0.560 [0.386;0.736] | 77.7% [69.1%;86.7%] | 86.8% [78.6%;94.8%] | 87.2% [75.8%;97.4%] | 68.2% [55.3%;82.2%] | 79.5% [70.6%;88.6%] | 79.0% [61.7%;95.7%] | 73.3% [62.9%;84.6%] | 84.2% [69.5%;96.9%] | 30 | 14 | 5 | 34 |
|  |  |  | HC vs. MCI vs. AD | 0.372 [0.249;0.497] | 58.7% [51.1%;66.8%] | 78.3% [72.8%;84.2%] | N/A | N/A | N/A | N/A | N/A | N/A | N/A | N/A | N/A | N/A |
| A7 | ADNI IR-SPGR (N=106) | ADNI IR-SPGR (N=46) | HC vs. MCI | 0.101 [-0.244;0.459] | 54.7% [38.3%;72.2%] | 60.2% [39.5%;80.2%] | 35.7% [11.1%;62.5%] | 73.7% [52.4%;94.1%] | 37.6% [9.3%;82.4%] | 72.1% [57.1%;85.0%] | 57.6% [18.8%;91.4%] | 53.4% [37.1%;71.5%] | 14 | 5 | 9 | 5 |
|  |  |  | HC vs. AD | 0.674 [0.382;0.934] | 83.2% [69.0%;95.8%] | 91.5% [80.0%;99.6%] | 76.9% [50.0%;100.0%] | 89.5% [75.0%;100.0%] | 82.1% [55.6%;100.0%] | 86.1% [70.6%;100.0%] | 88.0% [66.7%;100.0%] | 79.5% [60.0%;100.0%] | 17 | 2 | 3 | 10 |
|  |  |  | MCI vs. AD | 0.331 [-0.050;0.700] | 66.5% [47.5%;84.2%] | 75.8% [53.3%;93.2%] | 61.5% [33.3%;87.5%] | 71.4% [45.5%;92.9%] | 75.3% [46.4%;94.5%] | 56.7% [32.5%;84.0%] | 68,3% [37.9%;92.5%] | 65.0% [40.6%;88.1%] | 10 | 4 | 5 | 8 |
|  |  |  | HC vs. MCI vs. AD | 0.264 [0.030;0.507] | 51.9% [37.9%;66.8%] | 71.8% [60.7%;82.8%] | N/A | N/A | N/A | N/A | N/A | N/A | N/A | N/A | N/A | N/A |
| A8 | ADNI MPRAGE (N=423) | ADNI IR-SPGR (N=147) | HC vs. MCI | 0.242 [0.0649;0.0425] | 61.9% [52.4%;70.8%] | 68.8% [58.1%;78.9%] | 51.2% [36.2%;66.7%] | 72.6% [61.8%;83.3%] | 45.3% [29.6%;63.9%] | 77.1% [68.6%;85.0%] | 65.1% [48.7%;80.0%] | 59.8% [49.2%;71.4%] | 45 | 17 | 21 | 22 |
|  |  |  | HC vs. AD | 0.800 [0.678;0.911] | 89.2% [82.6%;95.4%] | 96.0% [92.2%;99.0%] | 83.3% [72.2%;94.6%] | 95.2% [89.5%;100.0%] | 91.6% [81.1%;100.0%] | 90.1% [83.7%;96.7%] | 94.6% [87.3%;100.0%] | 85.1% [76.3%;94.9%] | 59 | 3 | 7 | 35 |
|  |  |  | MCI vs. AD | 0.600 [0.421;0.764] | 80.0% [71.0%;88.0%] | 86.8% [78.4%;93.7%] | 81.0% [67.6%;92.3%] | 79.1% [65.9%;90.5%] | 84.6% [73.7%;93.2%] | 74.6% [59.0%;89.3%] | 79.5% [66.5%;90.7%] | 80.6% [67.0%;92.2%] | 34 | 9 | 8 | 34 |
|  |  |  | HC vs. MCI vs. AD | 0.457 [0.338;0.569] | 0.626% [0.553%;0.698%] | 79.8% [74.3%;85.1%] | N/A | N/A | N/A | N/A | N/A | N/A | N/A | N/A | N/A | N/A |
| A9 | ADNI IR-SPGR (N=147) | ADNI MPRAGE (N=423) | HC vs. MCI | 0.155 [0.047;0,267] | 56.0% [51.9%;60.5%] | 62.0% [55.8%;68.3%] | 24.8% [18.2%;31.7%] | 87.2% [81.9%;92.3%] | 46.2% [30.8%;64.6%] | 72.4% [69.3%;75.3%] | 66.0% [50.1%;80.5%] | 53.7% [50.0%;57.5%] | 130 | 19 | 109 | 36 |
|  |  |  | HC vs. AD | 0.711 [0,622;0.790] | 85.3% [80.8%;89.1%] | 94.5% [92.1%;96.7%] | 81.4% [74.3%;87.6%] | 89.3% [83.9%;94.1%] | 82.6% [74.3%;90.3%] | 88.5% [83.9%;92.4%] | 88.4% [82.2%;93.7%] | 82.8% [76.6%;88.4%] | 133 | 16 | 24 | 105 |
|  |  |  | MCI vs. AD | 0.531 [0.429;0.635] | 76.6% [71.5%;81.8%] | 82.8% [77.8%;87.4%] | 75.2% [67.5%;82.9%] | 77.9% [71.3%;84.7%] | 82.8% [76.9%;88.5%] | 68.9% [60.8%;77.8%] | 77.3% [70.2%;84.4%] | 75.9% [68.7%;83.2%] | 113 | 32 | 32 | 97 |
|  |  |  | HC vs. MCI vs. AD | 0.355 [0.290;0.416] | 55.4% [51.9%;59.0%] | 70.8% [67.7%;74.0%] | N/A | N/A | N/A | N/A | N/A | N/A | N/A | N/A | N/A | N/A |
| A10 | ADNI IR-SPGR and ADNI MPRAGE (N=379) | ADNI IR-SPGR and ADNI MPRAGE (N=164) | HC vs. MCI | 0.173 [0.001;0,354] | 58.6% [50.0%;67.6%] | 63.5% [53.5%;73.6%] | 52.8% [40.0%;66.7%] | 64.4% [52.6%;75.9%] | 39.7% [27.2%;55.1%] | 75.5% [66.4%;83.7%] | 59.7% [45.8%;73.5%] | 57.7% [46.7%;69.5%] | 38 | 21 | 25 | 28 |
|  |  |  | HC vs. AD | 0.784 [0.658;0.897] | 89.3% [83.0%;94.8%] | 95.7% [91.6%;98.8%] | 90.4% [81.0%;97.9%] | 88.1% [78.4%;96.6%] | 82.6% [70.1%;94.7%] | 93.6% [86.8%;98.7%] | 88.4% [78.9%;96.6%] | 90.2% [80.5%;97.9%] | 52 | 7 | 5 | 47 |
|  |  |  | MCI vs. AD | 0.603 [0.448;0.739] | 80.0% [72.4%;86.8%] | 88.0% [81.1%;93.7%] | 84.6% [74.1%;94.0%] | 75.5% [62.7%;85.9%] | 83.0% [73.8%;90.4%] | 77.6% [63.1%;91.0%] | 77.5% [66.5%;87.0%] | 83.1% [70.8%;93.5%] | 40 | 13 | 8 | 44 |
|  |  |  | HC vs. MCI vs. AD | 0.405 [0.291;0.512] | 60.3% [53.5%;66.8%] | 76.5% [71.0%;81.5%] | N/A | N/A | N/A | N/A | N/A | N/A | N/A | N/A | N/A | N/A |

*Footnote: PPV/NPV ‘prevalence’ are calculated with an MCI prevalence of 30.7% for the ‘HC vs. MCI’ classifiers; an AD prevalence of 38.5% for the ‘HC vs. AD’ classifiers; and an AD prevalence of 58.5% for the MCI vs. AD classifier (these correspond to the relative prevalence of the positive based on* prevalence estimates from the first visit in the clinical setting of 42.0% for HC, 18.6% for MCI, and 26.3% for AD) (47). *PPV/NPV ‘standard’ are calculated with a prevalence of 50% to allow comparison with other studies. Legend: CI = Confidence Interval; MCC = Matthew’s correlation coefficient; ROC AUC = Area under the receiver operating characteristic curve; BAC = Balanced Accuracy; Sens = Sensitivity; Spec = Specificity; PPV = Positive Predict Value; NPV= Negative Predictive Value; TN = True Negatives; FP = False Positives; FN = False Negatives; TP = True Positives.*

Supplementary Table 2 – *Classifier performance for ‘HC vs. AD’ classification, using morphometric and GT features for classifiers using ADNI and OASIS subjects.*

| Experiment | Training set | Testing set | MCC [CI: 95%] | BAC [CI: 95%] | ROC AUC [CI: 95%] | Sens [CI: 95%] | Spec [CI: 95%] | PPV (prevalence) [CI: 95%] | NPV (prevalence) [CI: 95%] | PPV (standard) [CI: 95%] | NPV (standard) [CI: 95%] | TN | FP | FN | TP |
| --- | --- | --- | --- | --- | --- | --- | --- | --- | --- | --- | --- | --- | --- | --- | --- |
| B6 | ADNI MPRAGE (N=194) | ADNI MPRAGE (N=84) | 0.810 [0.670;0.929] | 90.6% [84.0%;96.5%] | 97.8% [94.7%;99.7%] | 92.3% [82.9%;100.0%] | 88.9% [78.6%;97.6%] | 83.9% [70.8%;96.3%] | 94.9% [88.0%;100.0%] | 89.3% [79.5%;97.7%] | 92.0% [82.1%;100.0%] | 40 | 5 | 3 | 36 |
| B7 | OASIS (N=365) | OASIS (N=152) | 0.654 [0.456;0.843] | 78.9% [68.3%;90.5%] | 93.3% [87.7%;98.0%] | 60.0% [38.5%;83.3%] | 97.7% [94.7%;100.0%] | 94.2% [82.0%;100.0%] | 79.6% [71.1%;90.5%] | 96.3% [87.9%;100.0%] | 71.0% [60.6%;85.7%] | 129 | 3 | 8 | 12 |
| B8 | OASIS (N=517) | ADNI MPRAGE (N=278) | 0.739 [0.661;0.809] | 85.5% [81.2%;89.5%] | 96.7% [94.5%;98.4%] | 73.6% [66.0%;81.3%] | 97.3% [94.2%;99.4%] | 94.5% [87.7%;98.8%] | 85.5% [81.6%;89.5%] | 96.5% [91.9%;99.3%] | 78.7% [73.5%;84.2%] | 145 | 4 | 34 | 95 |
| B9 | ADNI MPRAGE (N=278) | OASIS (N=517) | 0.674 [0.585;0.759] | 88.5% [83.6%;92.9%] | 94.0% [90.0%;97.2%] | 85.1% [75.8%;93.3%] | 92.0% [89.5%;94.5%] | 86.9% [81.9%;91.4%] | 90.8% [85.5%;95.8%] | 91.4% [87.8%;94.4%] | 86.1% [78.7%;93.4%] | 414 | 36 | 10 | 57 |
| B10 | ADNI MPRAGE and OASIS (N=295) | ADNI MPRAGE and OASIS (N=127) | 0.842 [0.741;0.924] | 92.1% [87.2%;96.4%] | 96.8% [93.7%;99.2%] | 91.5% [83.9%;98.2%] | 92.6% [85.7%;98.4%] | 88.6% [78.6%;97.5%] | 94.6% [89.5%;98.9%] | 92.6% [85.5%;98.4%] | 91.6% [84.2%;98.2%] | 63 | 5 | 5 | 54 |

*Footnote: PPV/NPV ‘prevalence’ are calculated with an AD prevalence of 38.5% (this corresponds to the prevalence of AD relative to HC based on* prevalence estimates from the first visit in the clinical setting of 42.0% for HC and 26.3% for AD) (47). *PPV/NPV ‘standard’ are calculated with a prevalence of 50% to allow for comparison with other studies. Legend: CI = Confidence Interval; MCC = Matthews correlation coefficient; ROC AUC = Area under the receiver operating characteristic curve ; BAC = Balanced Accuracy; Sens = Sensitivity; Spec = Specificity; PPV = Positive Predict Value; NPV= Negative Predictive Value; TN = True Negatives; FP = False Positives; FN = False Negatives; TP = True Positives.*

Supplementary Table 3 - *Relative contribution of features for the HC vs. AD classifier trained and tested both on IR-SPGR and MPRAGE scans (Experiment A5), segregated by brain region and type*

| Type/Region | Central | Cingulate | Frontal | HPC | Insula | Occipital | Occipito-Temporal | Parietal | Temporal | Basal Ganglia | Corpus Callosum | CSF | Other |
| --- | --- | --- | --- | --- | --- | --- | --- | --- | --- | --- | --- | --- | --- |
| Volume | 0.3% | 2.7% | 2.6% | 31.4% | 0.0% | 0.7% | 0.9% | 1.3% | 2.4% | 2.1% | 0.1% | 5.6% | 3.3% |
| Surface Area | 1.5% | 1.3% | 0.6% | 0.4% | 0.0% | 0.0% | 0.2% | 0.5% | 0.7% | n/a | n/a | n/a | 0.0% |
| Cortical Thickness | 1.2% | 1.8% | 2.4% | 3.6% | 0.7% | 0.6% | 1.0% | 3.1% | 5.7% | n/a | n/a | n/a | 1.0% |
| StDev Thickness | 1.5% | 1.7% | 1.3% | 0.1% | 1.5% | 0.1% | 0.2% | 0.3% | 1.1% | n/a | n/a | n/a | n/a |
| Mean Curvature | 0.2% | 0.7% | 0.2% | 0.4% | 0.1% | 0.0% | 0.1% | 0.5% | 1.2% | n/a | n/a | n/a | n/a |
| Gaussian Curvature | 0.3% | 0.7% | 0.9% | 0.5% | 0.0% | 0.0% | 0.1% | 0.6% | 0.8% | n/a | n/a | n/a | n/a |
| Folding Index | 0.8% | 1.3% | 0.2% | 0.1% | 0.1% | 0.1% | 0.1% | 0.1% | 0.5% | n/a | n/a | n/a | n/a |
| Curvature Index | 0.2% | 0.9% | 0.3% | 0.2% | 0.0% | 0.0% | 0.1% | 0.2% | 0.0% | n/a | n/a | n/a | n/a |

*Footnote: StDev Thickness = Standard deviation of thickness; HPC = Hippocampus; CSF = Cerebrospinal fluid.*

Supplementary Table 4 - *Relative contribution of features for the HC vs. AD classifier trained and tested both on ADNI subjects with MPRAGE scans and OASIS subjects (Experiment B5), segregated by brain region*

| Type/Region | Central | Cingulate | Frontal | HPC | Insula | Occipital | Occipito-Temporal | Parietal | Temporal | Basal Ganglia | Corpus Callosum | CSF | Other |
| --- | --- | --- | --- | --- | --- | --- | --- | --- | --- | --- | --- | --- | --- |
| Volume | 1.7% | 3.6% | 2.6% | 22.3% | 0.2% | 0.5% | 0.7% | 1.2% | 2.7% | 4.5% | 0.4% | 4.3% | 1.8% |
| Surface Area | 1.1% | 1.3% | 1.0% | 0.2% | 0.2% | 0.2% | 0.1% | 0.1% | 0.8% | n/a | n/a | n/a | 0.0% |
| Cortical Thickness | 1.5% | 2.4% | 1.2% | 3.1% | 0.8% | 0.3% | 0.6% | 2.6% | 4.6% | n/a | n/a | n/a | 0.3% |
| StDev Thickness | 2.9% | 2.4% | 1.8% | 0.4% | 1.5% | 1.1% | 0.6% | 1.1% | 2.1% | n/a | n/a | n/a | n/a |
| Mean Curvature | 0.2% | 0.4% | 1.2% | 0.3% | 0.1% | 0.1% | 0.5% | 1.3% | 2.2% | n/a | n/a | n/a | n/a |
| Gaussian Curvature | 0.4% | 1.4% | 1.1% | 0.2% | 0.0% | 0.1% | 0.4% | 0.3% | 0.4% | n/a | n/a | n/a | n/a |
| Folding Index | 0.6% | 0.6% | 0.5% | 0.2% | 0.0% | 0.2% | 0.3% | 0.7% | 0.6% | n/a | n/a | n/a | n/a |
| Curvature Index | 0.4% | 0.7% | 0.8% | 0.1% | 0.0% | 0.1% | 0.4% | 0.3% | 0.3% | n/a | n/a | n/a | n/a |

*Footnote: StDev Thickness = Standard deviation of thickness; HPC = Hippocampus; CSF = Cerebrospinal fluid.*
